# Membrane Technologies for Nitrogen Recovery from Waste Streams: Scientometrics and Technical Analysis

**DOI:** 10.3390/membranes13010015

**Published:** 2022-12-22

**Authors:** Raed A. Al-Juboori, Muayad Al-Shaeli, Saif Al Aani, Daniel Johnson, Nidal Hilal

**Affiliations:** 1 NYUAD Water Research Centre, New York University, Abu Dhabi Campus, Abu Dhabi P.O. Box 129188, United Arab Emirates; 2 Department of Engineering, University of Luxembourg, 2, Avenue de l’Université, L-4365 Esch-sur-Alzette, Luxembourg; 3 The State Company of Energy Production-Middle Region, Ministry of Electricity, Baghdad 10013, Iraq

**Keywords:** nitrogen recovery, waste stream, membrane technologies, scientometrics, hybrid systems

## Abstract

The concerns regarding the reactive nitrogen levels exceeding the planetary limits are well documented in the literature. A large portion of anthropogenic nitrogen ends in wastewater. Nitrogen removal in typical wastewater treatment processes consumes a considerable amount of energy. Nitrogen recovery can help in saving energy and meeting the regulatory discharge limits. This has motivated researchers and industry professionals alike to devise effective nitrogen recovery systems. Membrane technologies form a fundamental part of these systems. This work presents a thorough overview of the subject using scientometric analysis and presents an evaluation of membrane technologies guided by literature findings. The focus of nitrogen recovery research has shifted over time from nutrient concentration to the production of marketable products using improved membrane materials and designs. A practical approach for selecting hybrid systems based on the recovery goals has been proposed. A comparison between membrane technologies in terms of energy requirements, recovery efficiency, and process scale showed that gas permeable membrane (GPM) and its combination with other technologies are the most promising recovery techniques and they merit further industry attention and investment. Recommendations for potential future search trends based on industry and end users’ needs have also been proposed.

## 1. Introduction

The dynamic equilibrium of the global cycle of nitrogen has recently been experiencing a concerning disturbance. The last century has witnessed an increase in anthropogenic nitrogen that doubled the global cycle of reactive nitrogen [1]. The rise in world population contributes to both the growing need for increasing nitrogen fertilizers required for meeting concomitant climbing food demands [2] and the rising discharge of anthropogenic nitrogen. This presents dual challenges of meeting supply demands and protecting the environment from detrimental impacts of nitrogen contamination (e.g., eutrophication). Currently, nitrogen fertilizers are produced through the Haber Bosch process. This process is known to be energy-intensive process and environmentally unfriendly. The process consumes about 35–50 MJ/kg N [3] and this on a bigger scale translates into 1–2% of total world energy [4]. The production of one ton of ammonia (NH_3_) fertilizer requires 949 m^3^ of natural gas and emits 1.6 tons of carbon dioxide. The pollution brought by nitrogen and phosphorous has also been translated into cost figures by some studies. For example, Sutton et al. [5] quoted a European study concerning the annual cost of nitrogen pollution that was estimated to be €75–485 billion based on the 2008 nitrogen discharged into water systems and emitted to the air. This is a clear indication that we need to improve the processes that involve production and discharge of nitrogen. This issue should be tackled in such a way where a circular sustainable economy takes over and linear economy practices are phased out.

The other dimension of the nitrogen issue is the way it is currently removed from wastewater. Commonly, reactive nitrogen in wastewater is converted to mostly N_2_ that gets released into the atmosphere, and the rest to biomass through a range of microbial processes. Biological nitrogen removal processes are well known in the industry, such as nitrification/denitrification with different air availability, anaerobic ammonium oxidation (anammox), CANON, or their combinations [1]. These processes have been serving the great purpose of protecting the environment from harmful domestic and industrial discharges. However, these processes require a large amount of energy to operate given the large volume of wastewater. There is a consensus in the literature that the aeration required for the biological removal of nitrogen takes up to 50–60% of the total energy required in wastewater treatment plants [6,7,8]. The international energy agency (IEA) reported that wastewater treatment consumes about 200 TWh, which makes up 2% of world energy consumption [9]. Based on these figures, the aeration required for nitrogen removal consumes roughly 1% of world energy. IEA has also indicated that there are great opportunities for energy saving in wastewater through recovery and process improvement. In this regard, the recovery of nitrogen and other resources has become a necessity to maintain the sustainability of the wastewater treatment industry. Nitrogen recovery can reduce the energy requirement of aeration in the first place, in addition to the generation of useful products, such as fertilizers, and the reduction of greenhouse gas emissions (e.g., N_2_O).

The benefits of nitrogen recovery have led to the emergence of a large number of innovative technologies. These technologies include electrochemical and bio-electrochemical processes, conventional stripping process (ammonia air stripping), struvite precipitation process, and membrane separation processes [1,10,11]. Combinations of these technologies have also been reported in the literature in various degrees of complexity. Among all these technologies, membrane separation processes stand out due to their maturity, practicality, and relatively low energy requirement of the technology. There are few literature review papers concerning resource recovery from wastewater with the aid of membrane technologies. However, these papers either have a broad scope in terms of recovered resources or a very narrow scope to only a single membrane technology [12,13]. This work attempts to find a balance between these two approaches in terms of the scope of the technologies and recovered resources. It focuses only on nitrogen recovery from different wastewater streams using various membrane technologies. Additionally, the work also presents bibliometric analyses of the literature body for nitrogen recovery specifically with membrane technologies to understand the evolvement of the literature trends and identify the key players in this research area. Such analyses have not previously been reported.

## 2. Literature Analysis

Bibliometric analysis is a useful tool for understanding the literature. Such a practice enables readers to have an overview of the development of the topic of interest and understand the dynamicity of the research being conducted. More importantly, it helps in identifying the emerging research trends and the possible synergies between proposed solutions for research. A critical scientometric analysis of the literature was conducted using information extracted from credible sources such as the Web of Science and Scopus. Similar approaches were reported to be useful for analyzing literature pertaining to the water research field such as the study conducted on forward osmosis applications in desalination and wastewater [14] and the adsorptive membrane technologies for resources recovery [15].

**Figure 1:**
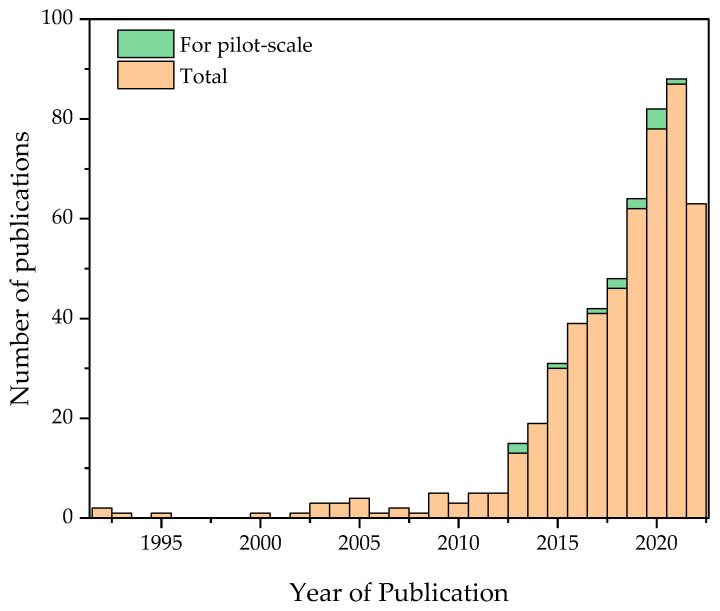
Publications number over recorded years of publication.

The data extraction was based on a search performed on the Web of Science on 05/10/2022 using the following terms as keywords: “*nitrogen recovery*”, “*membrane technology*” and “*wastewater*”. Only documents published in the English language were considered in the analysis. The search returned a total number of 543 documents, of which there are 448 research articles, 55 review articles, 6 book chapters, 5 early access documents, 27 conference proceedings, and 2 meeting notes. There was no filter applied for the publication period, and the earliest record found dates back to 1992. The interest in recovering nitrogen from wastewater with the aid of membranes saw the light that year through the work of Voortman and co-workers on recovering calcium nitrate from an aqueous effluent rich with ammonium nitrate using an electrochemical membrane cell [16]. Their work was motivated by the United States Environmental Protection Agency (USEPA) Resource Conservation Recovery act authorized in 1986. Although the approach proposed by the Voortman team was attractive, especially as it was proven to be useful for the recovery of other materials, it did not capture the attention of researchers. The years after this work witnessed attempts focused on membrane applications for water reclamation, improving nutrient removal, and concentrating nutrients for possible applications in agricultural fields. Examples of these studies are reverse osmosis (RO) trials for nitrogen removal from wastewater effluent in Norway [17], a pilot study for membrane bioreactor (MBR) for nitrogen removal from opto-electronic industry wastewater [18], and farm-scale of microfiltration (MF) and RO for nitrogen removal from sow slurry in Belgium [19]. Other filtration technology combinations have also been proposed in the literature, such as the work of Jonas and Daniel who suggested the spread of the MBR-RO concentrate, which is rich in nitrogen and phosphorous, onto agricultural fields [20]. From 2008 onward, this research area picked up and the interest has been growing significantly since then, as shown in Figure 1. Nitrogen recovery is expected to attract even more attention with the current situation of the Russia-Ukraine war and the resultant trade restrictions and international sanctions. According to a recent publication by the Food and Agriculture Organization of the United Nations (FAO), Russia was globally the top exporter of nitrogen fertilizers in 2021 [21]. Additionally, the increasing demands for fertilizers and the commitment to reducing emissions will also motivate researchers to increase their research activities in this field. Despite the impressive increase in the number of research publications, there has been only little effort dedicated to the translation of the research ideas to pilot- or large-scale applications. In fact, out of the 543 publications, there are only 10 publications reported on the pilot-scale membrane nitrogen recovery unit, and these are seen in the histogram stack of Figure 1.

**Figure 2:**
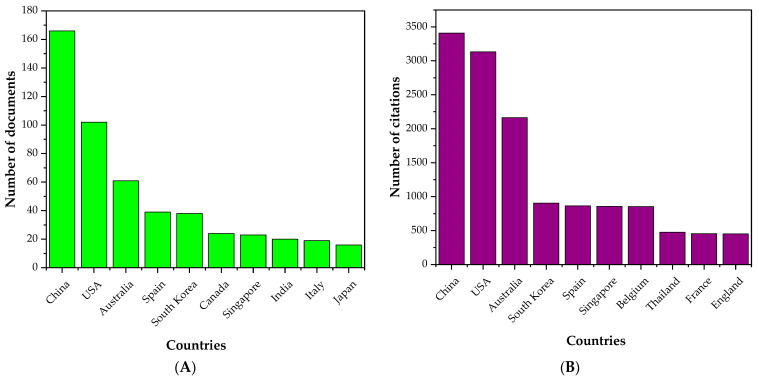
Top 10 countries in (**A**) publications number and (**B**) citations for research documents related to membrane application for nitrogen recovery from wastewater.

To map out the research activities in the field of membrane application for nitrogen recovery, the top 10 countries in terms of the number of publications and citations were identified and the results are presented in Figure 2. China, the USA, and Australia are the top three countries in both the number of publications and citations. This could be due to the fact that these three countries are the biggest agricultural countries worldwide, and this has driven the research toward nutrient recovery. The rest of the top 10 countries is dominated by European and Southeast Asian countries.

**Figure 3:**
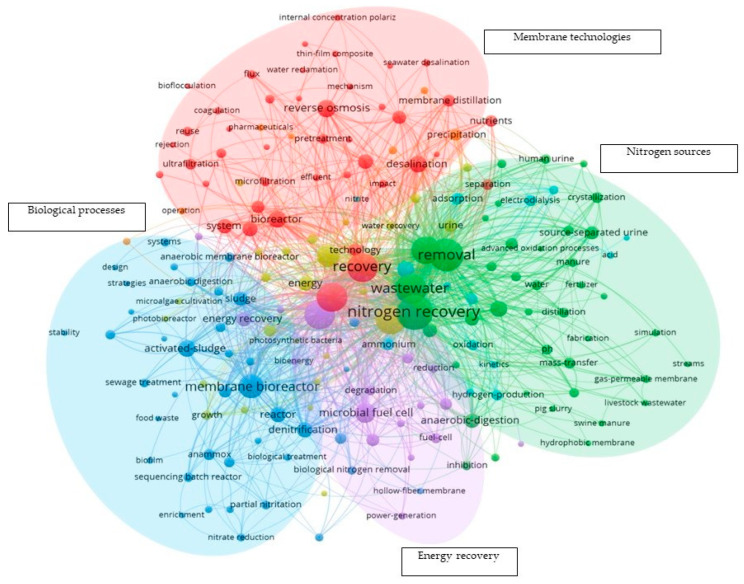
Keywords co-occurrence map.

For a deeper analysis of the literature data, VOSviewer has been used for constructing maps for keywords frequency occurrence, researchers’ collaboration across countries, and the use of produced research documents in different parts of the world. Several criteria were applied for constructing these maps. For the keyword occurrence map, a minimum number of 5 occurrences is required for a certain keyword to be counted. For countries to appear on co-authorship and citation maps, they need to have at least 1 document and 1 citation, respectively. In addition, due to the use of different wording, structures, and acronyms for the same terms, a thesaurus file was created where keywords with the same meaning were replaced by one main keyword as shown in Table 1. Keyword maps help in identifying trends and themes in the research area being studied. Figure 3 shows the keywords occurrence map. The size of the bubbles reflects the frequency of the keywords and the connecting lines represent the co-occurrence of the keywords together. There are five distinct clusters marked with different colors. Out of these five clusters, four research themes can be identified. These are biological processes, membrane technologies, nitrogen resources, and energy recovery. Membrane technologies play a significant role in nitrogen recovery research as it occupies one of the main four themes and it appears in other themes as well. For example, membrane bio-reactor has the biggest bubble in the biological process cluster. Similarly, hollow fiber membrane and gas permeable membrane (GPM) keywords appear in the energy recovery and nitrogen resources clusters. Almost all kinds of membrane technologies are present in the keyword occurrence map such as RO, Ultrafiltration (UF), MF and Membrane distillation (MD). There are other membrane technologies such as Forward Osmosis (FO) and GPM that are not shown in Figure 3 due to the presentation limitation, but they have been spotted within the pool of the keywords that passed the threshold of 5 times occurrence in the extracted data.

**Figure 4:**
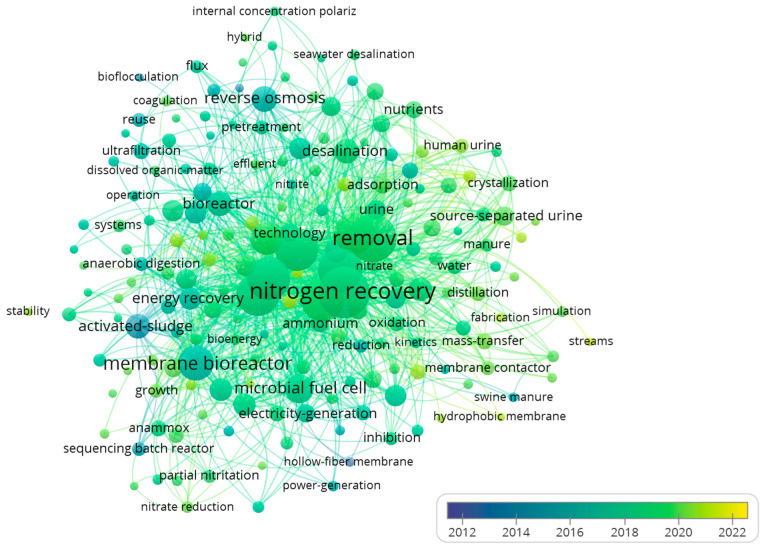
Keywords occurrence change in the past 10 years.

The historical change in the keywords for the past 10 years has also been tracked and the results are depicted in Figure 4. There is a shift in the research focus from recovering water, concentrating nutrients, and removing organics to exploring new waste streams for nitrogen recovery, utilization of advanced software tools, refining the recovered materials, and improving membrane surface characterization. This is marked by the change in the keywords across the selected timeline from activated sludge, membrane bio-reactor, and reverse osmosis to urine, streams, crystallization, simulation, and hydrophobic membrane. This is evident in recent research publications on nitrogen recovery from wastewater. Recently, a large number of publications have been focusing on topics such as nitrogen recovery from urine [22,23], improving membrane properties and design for nitrogen recovery [24], applying modeling for studying nitrogen recovery [25] and investigating the quality of the produced ammonium salts [26].

**Figure 5:**
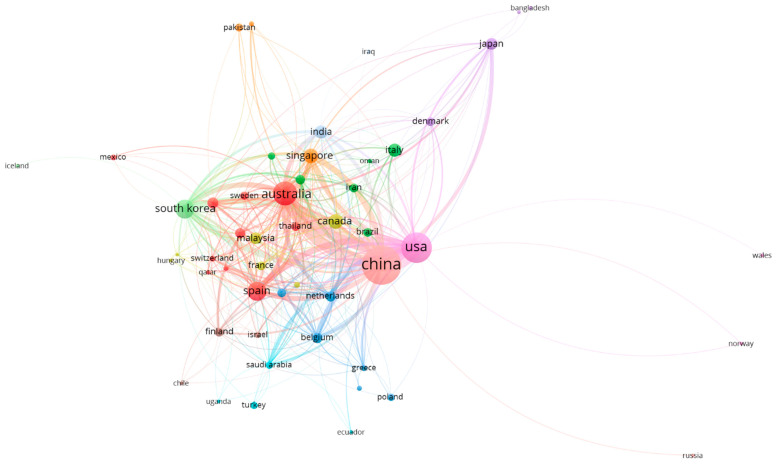
A map of the most cited countries based on the number of documents.

**Figure 6:**
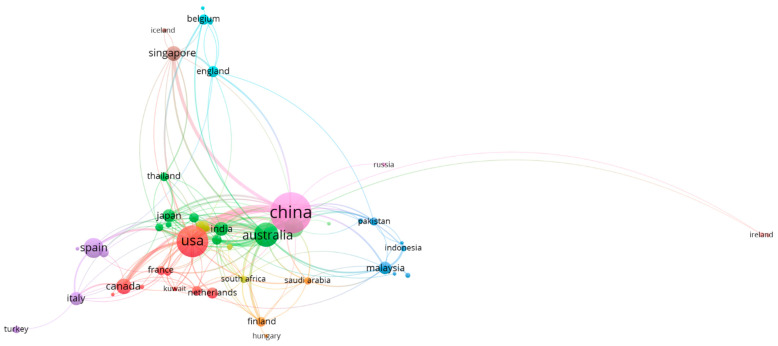
Co-authorship map based on the number of documents.

Figure 5 and Figure 6 show the country maps based on citation and number of co-authored documents, respectively. Here, the thickness of the lines connecting the bubbles demonstrates the strong collaboration and co-citation. As stated earlier, China, the USA, and Australia dominate this research field and there are solid collaborations between these countries. Some other countries such as Spain, South Korea, and Singapore also have a decent share of the knowledge pool. The European countries have formed most of their collaborations with the USA and Australia, while East and Southeast Asian countries’ collaborations were mainly with China. Countries from South Asia and the Middle East seem to have their collaboration spread out across all regions. These analyses affirm that membrane technologies had and will continue to have a significant impact on the nitrogen recovery field. A study conducted by van der Hoek et al. [27] for evaluating the tested technologies for nitrogen recovery from wastewater showed that membrane technologies scored highly between positive and very positive scale for the examined assessment criteria of sustainability, products readiness for market release, maturity and the concentration range of nitrogen. The following sections will critically review the outcomes reported for the application of membrane technologies for nitrogen recovery from different waste streams.

## 3. Nitrogen Recovery Waste Streams

This section is concerned with discussing the potential nitrogen recovery waste streams. The various streams that have proven to be valuable sources of nitrogen recovery are presented in Table 2. Several factors affect nitrogen recovery from wastewater, such as the concentration of reactive nitrogen (i.e., NH_4_^+^), solids concentration in the stream, and other characteristics including pH level and the concentration of organic and inorganic constituent contaminants. The organic concentration is expressed commonly in chemical oxygen demand (COD) units or total organic carbon (TOC). Total dissolved solids (TDS) or conductivity can be used as an estimation for the inorganic content of wastewater. The concentration of nitrogen determines the feasibility of the recovery process and the requirement for pre-treatment steps. The solids can negatively affect nitrogen recovery as they could damage the membrane surface, block membrane pores or even provide surfaces for ammonia adsorption [23,26]. The pH level influences the available nitrogen species, the charge of the membrane surface, and the chemistry of the pollutants. The most important effect is the change in ammonia/ammonium fraction, which can be calculated using Equations (1) and (2) at 20 °C [28]. The desired pH for nitrogen recovery depends on the applied membrane technology. For GPM technology, an alkali pH range is preferred as nitrogen is recovered in the form of gas (i.e., NH_3_). The case is different for pressured-driven membranes where nitrogen is recovered through concentrating NH_4_^+^ in the feed solution. The pH was also found to affect the charge of the membrane surface, and hence its rejection capacity for ammonium [29]. Similarly, pH can affect organic and inorganic membrane fouling [30]. The inorganic pollutants can affect the efficiency of nitrogen recovery through their involvement in fouling formation. However, this depends on the treatment and operational conditions. For example, the presence of bicarbonate in livestock wastewater aided nitrogen recovery with GPM due to their alkaline nature that promotes the conversion of NH_4_^+^ to NH_3_ [31]. In contrast, bicarbonate induces inorganic fouling in other membrane technologies such as MD by inducing the formation of the most common inorganic foulant, calcium carbonate (CaCO_3_) [32]. Other inorganic constituents, such as metals and metalloids, can precipitate on membrane surfaces in the form of salts (e.g., Mg(OH)_2_) depending on their solubility in the applied treatment conditions [33]. Organics present in the waste stream are in general troublesome for nitrogen recovery as they could cause fouling of all membrane technologies [10,34], and may compete with NH_3_ transfer in the case of GPM if they are present in the volatile form. It should be mentioned though that the tolerance of membranes to organic fouling depends on the used technology. Non-pressurized membranes such as GPM were found to maintain their nitrogen recovery performance even with the presence of a high concentration of humic acid in the range of 3–6 g/L [31].

(1) $$pK_{NH_{4}^{+}}=\frac{2755.16}{T}$$

(2) $$\left[NH_{3}\right]=\frac{\left[NH_{4}^{+}\right]}{10^{pK_{NH_{4}^{+}}-pH}+1}$$

**Figure 7:**
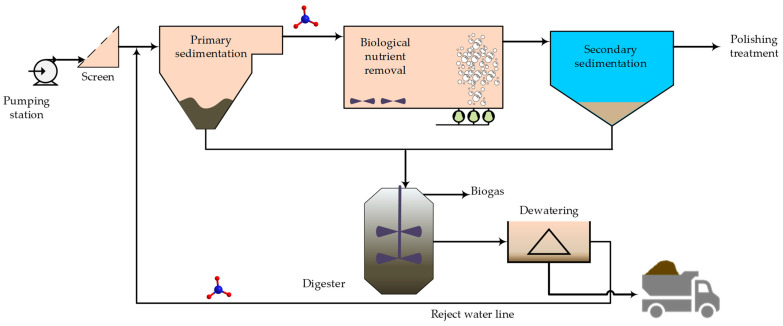
A simplified scheme of a typical wastewater treatment plant.

A recent systematic literature survey focusing on the sustainability of nitrogen recovery from waste showed that domestic wastewater is the most explored stream for this purpose [35]. This is due to the volume of the stream and the stringent discharge and emission limits imposed upon wastewater treatment plants. Figure 7 shows a schematic for a typical wastewater treatment train. Researchers envisaged recovery opportunities in two main locations on the wastewater treatment line. Some suggested the use of filtration preconcentration for retaining nutrients in the primary effluent [33,36,37]. Others suggested ammonia extraction from the reject water line after the digester [26,38,39]. These two locations are marked in Figure 7 by the ammonia molecule symbol. The concentration of nitrogen and other parameters varies along the treatment line of wastewater. The nitrogen range of domestic wastewater in Table 2 represents the low range which is present in the influent and the high range which is present in the concentrated streams such as the reject water line, known also as centrate. It is worth mentioning that the concentration of nitrogen in the rejected water could vary seasonally depending on the efficiency of nitrogen removal in the biological treatment [40].

Urine makes 80% of the nitrogen and 50% of the phosphorous of wastewater, although its contribution to wastewater volumetric flow is only about 1% [41]. This highlights how nutrient-rich this stream is, and emphasizes the potency of urine separation as a viable way of reducing the load in wastewater, which affords great opportunities for nutrient recovery given the low solids content. It is noteworthy that studies dealt with urine streams in two main forms; fresh urine and hydrolyzed urine. The difference between these two streams is that the nitrogen in fresh urine exists mainly as urea (90%) at low pH of 6. Once the urine encounters Urease, which is an abundant bacterial and fungal enzyme, the urea is converted to ammonia and bicarbonate raising the pH to 9. This form of urine is referred to as hydrolyzed urine [42]. The conversion of urea to NH_4_^+^, NH_3,_ and HCO_3_^−^ increases pH, and this, in turn, affects the balance between NH_4_^+^ and the soluble NH_3_ in urine causing the latter to release into the atmosphere producing an unpleasant odor [43]. The unpleasant smell is not the only problem with hydrolyzed urine, the formation of precipitants such as struvite, potassium struvite, and hydroxyapatite is another technical problem for storing and transporting urine as they could block drains and attach to the walls in the tanks and urinal traps [43]. It is noteworthy that these precipitates are considered valuable fertilizers, however, their formation needs to be controlled to take place in appropriate recovery units. To overcome the odor and scaling problems of hydrolyzed urine, stabilization techniques are normally implemented such as controlling urease enzyme activation through pH manipulation by adding Ca(OH)_2_ to the collection tank [44], using enzyme inhibitors, or applying electrochemical techniques [45]. Urease has an optimum pH range of 6.8–8.7 [46].

Manure is defined as a mixture of excrement and urine produced from livestock with or without litter [47]. Manure is defined as slurry when the solid contents range between 4% and 20% [48]. This waste stream is produced in large quantities around the world. According to recent European statistics, there were about 1.4 billion tons of animal farm manure produced annually in the European Union and the United Kingdom for the period between 2016–2019 [47]. The spread of manure slurry directly or after simple processing onto fields is an old farming practice. However, due to environmental and health risks, such practices have been regulated and defined limits of applications have been implemented. For instance, the European Nitrate directive set a ceiling of 170 kg N/ ha. per year for manure spreading [49]. This emanates from the risks associated with the application of surpluses of nutrients that can leach to groundwater [50], the high concentration of heavy metals stemming from the use of animal feed supplements [51], and the presence of antibiotics used for maintaining animal health [52]. For these reasons, extracting nutrients such as nitrogen using membrane separation has emerged as a solution for benefiting from manure while maintaining the quality of the extracted products. It can be seen in Table 2 that manure slurry contains considerable nitrogen concentration falling between nitrogen ranges of wastewater and urine. However, it contains much higher solids and organic and inorganic pollutants compared to these streams. Therefore, solids separation techniques such as filter press, cyclones, screw presses are normally applied prior to membrane filtration step [19].

The idea of wastewater source separation was promulgated by experts calling for a change in the linear economy approach of wastewater management (collect-treat-dispose). This approach has been proven to be costly from an environmental and energy consumption perspective. Source separation has been envisaged as a way for sustainable management of wastewater with maximizing energy and nutrient recovery at the source [53]. The separation is based on segregating household wastewater into blackwater (mainly toilet flushing), urine, and greywater (kitchen and shower wastewater). The promise behind this segregation is to achieve waste streams that are rich in nutrients for subsequent recovery, as well as to allow localized recycling of less contaminated water for non-potable use, such as toilet flush water. Separation does not only decrease the dilution effect on nutrients, but it also helps in developing customized treatment solutions targeted toward certain classes of pollutants that are known to be more concentrated in one of the three streams compared to the others. For instance, greywater contains higher heavy metals compared to urine and blackwater [54]. Similarly, blackwater contains the highest pathogen share in wastewater [55], while most pharmaceuticals and hormones found in wastewater come from urine [56]. Northern Europe has been one of the most advocating regions for source separation of wastewater [57]. Although the focus of source separation has historically been directed towards urine as dry urine toilets were available since the 1970s [58]. Despite the extensive efforts for the implementation of source separation represented by pilot trial projects in countries such as Sweden and Switzerland, the real applications of source separation are still confined to small rural areas outside the service coverage of existing wastewater jurisdictions [59]. Although blackwater and urine are small streams in volume, they are still important for sparsely populated countries, such as Finland and Sweden, and tourist areas [55,59,60]. It is noteworthy that due to the high carbon content in blackwater, it is more favorable to energy production (anaerobic methane generation) than direct nitrogen recovery. However, nitrogen recovery can still be applied after anaerobic digestion.

**Figure 8:**
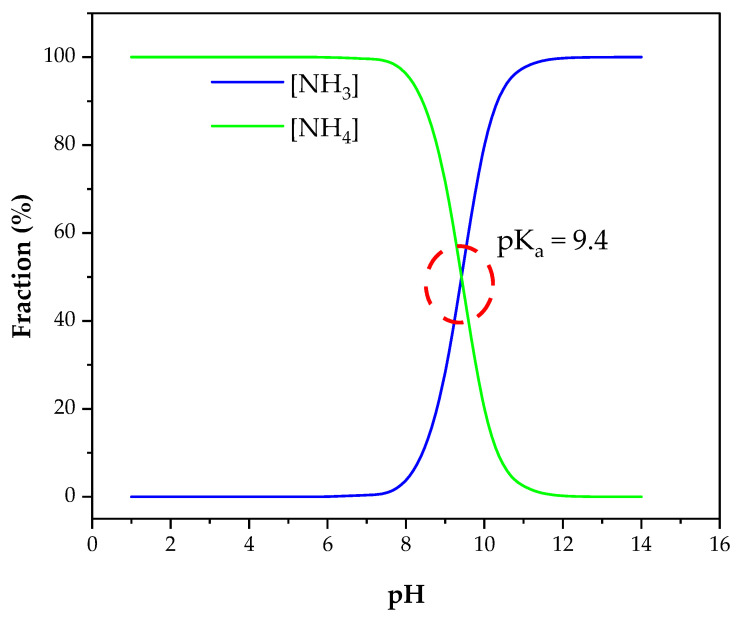
Ammonia/Ammonium speciation based on pH level at 20 °C.

It has been estimated that about 17% of global food production is wasted [61], and this accounts for 1.6 billion tonnes of food waste yearly [62]. The most common practice for food waste treatment is anaerobic digestion. However, the production of ammonia in high concentrations from organic nitrogen materials can inhibit anaerobic microbes such as methanogens [63]. So, this necessitates the need for an effective nitrogen removal technique. This need has driven the research in nitrogen recovery from food waste for the dual benefits of improving waste digestion and energy production and recovering valuable products. Water could also be recovered when biological membrane techniques such as anaerobic membrane bioreactor (AnMBR) are used [64]. It can be noticed though that the solid content of the stream is the highest compared to other streams and the pH is lowest suggesting that direct nitrogen recovery can be challenging for membrane technologies. Some studies suggest the simultaneous application of digestion and membrane separation for food waste treatment and nitrogen recovery [65]. However, the compatibility of the digestion environment and ammonia recovery conditions is hard to tune. It was elucidated in Figure 8 that a pH of >9 is required for ammonia recovery, whereas a neutral pH level was found to be optimal for food waste digestion [66].

Aquaculture is defined as the industry of producing seafood and aquatic plant cultivation for food and non-food demands [67]. This industry discharges large amounts of wastewater. Depending on the system configuration, aquaculture consumes between 0.3 m^3^/kg production per year for most efficient systems (super intensive recirculation aquaculture) and 45 m^3^/kg production per year for least efficient systems (extensive pond farming) [68]. In the resources’ recovery context, reclaiming water for reuse in aquaculture seems to be more important than nitrogen recovery given its small concentration in this stream. Nitrogen recovery can be a useful by-product of water reclamation. A recent study by Teoh et al. [69] showed that MD could be used for producing clean water as well as a reject highly concentrated with nutrients such as ammonia, phosphorous and potassium that can be used as a liquid fertilizer. Although with the pH range of aquaculture (6–8), some of the dissolved ammonia may convert to the gaseous phase and escape to the permeate side along with volatile carbon in the MD process.

The slaughterhouse industry consumes 29% of the used freshwater in agriculture, which has been estimated to be 70% of the global freshwater consumption [70,71]. This large amount of water is then turned into wastewater with high content of solids, organic pollutants, and nutrients. The most common nitrogen form in slaughterhouse wastewater is ammonia [72]. The highest ammonia concentration is found in the rendering plant of the slaughtering process train. Nitrogen is commonly removed from this stream through the nitrification/denitrification process. Since slaughterhouse wastewater is rich in fats and organic matter, it can be utilized for biogas production. As mentioned earlier, ammonia inhibits microbes involved in the conversion of organic materials into methane. Hence, recovering ammonia has an added advantage on top of fertilizer production and reduction of nitrogen concentration in the effluent. Using membrane technologies for ammonia recovery from slaughterhouse wastewater can be a daunting task due to the presence of difficult contaminants such as high concentrations of proteins, fats, and grease that can easily and quickly foul the membrane [72]. Vigorous pre-treatment processes needed to be implemented to insure high removal of these contaminants ahead of the membrane process. Considering the concentration of ammonia in slaughterhouse wastewater, recovering nitrogen alone cannot make the process economically feasible. This may be the reason behind the lack of investment in developing nitrogen recovery processes for slaughterhouse wastewater [73]. For nitrogen recovery to be attractive to investors, it has to be presented as a solution for reducing energy requirements in nitrification-denitrification, improving biogas production, and producing fertilizers.

Landfill leachate is the percolated liquid stream produced from the decomposition of landfill wastes [74]. This stream is characterized by its high content of toxic materials, such as heavy metals and polyaromatic hydrocarbon [74,75]. This stream has a high nitrogen content, as shown in Table 2. Landfill leachate is normally directed to a wastewater collection system. Biological removal of nitrogen from this stream is hard due to high salinity and lack of electron donors, especially in the stabilized landfills [76]. Therefore, removing nitrogen onsite makes practical and economic sense. The low solids content and relatively high pH makes landfill leachate one of the most promising waste stream for nitrogen recovery using membrane technology.

## 4. Pressure-Driven Membrane Processes

Pressure-driven membranes are an established technology that has been utilized in many industrial applications including water and wastewater treatment, desalination, pharmaceutical industries, and food production due to their high separation performance [1,2]. Pressure-driven membrane processes include MF, UF, nanofiltration (NF), and RO [118,119]. The membrane pore size and pressured requirement differ in these technologies. The nominal pore size of MF, UF, and NF is 0.1 μm, 0.01 μm and 0.001 μm, respectively, while the RO membrane is regarded as nonporous [120]. The operating pressure varies depending on the quality of the feed stream. For wastewater streams such as animal waste, the transmembrane pressure for MF, UF, NF and RO are in the ranges of 100–180 kPa, ≤800 kPa, 350–3000 kPa, and 3.5–6.5 MPa [121]. The operating pressure of specially designed RO membrane can reach up to 150 MPa, but the economics of the process might not be feasible if we consider nitrogen recovery.

Pressure-driven membranes are normally differentiated based on the contaminants they reject. MF can remove particles larger than their pore size as well as algae and bacteria, small colloids and viruses are removed by UF, whereas NF removes dissolved organic matter and multivalent ions. RO can even remove monovalent species. In the context of nitrogen recovery, the rejection mechanisms of different nitrogen species are important. The dominant rejection mechanism in MF and UF is straining and this means that molecules/ions with diameters higher than the nominal pore size can be retained. The hydrated radius of the ammonium ion is 0.25 nm [122], which is much smaller than the nominal pore size of MF, UF, and even NF. This means that ammonium can easily pass through MF and UF, but it may react with phosphorous and magnesium forming struvite in the feed stream. Hence, MF and UF are normally applied as pre-treatment for NF and RO application for nitrogen recovery. Their capacity to remove solids, microbes and viruses helps reduce fouling and improves the quality of the recovered water. Some waste streams contain considerably high solids contents such as manure slurry and food waste that makes direct use of MF and UF with such wastes unfeasible. In these cases, other solid separation processes are utilized as pretreatment for MF and UF. Some studies found that nitrogen retention can be improved by circulation, however, this is not suitable for processes with short retention times and also nitrogen could be lost to biological activities and evaporation [79].

**Figure 9:**
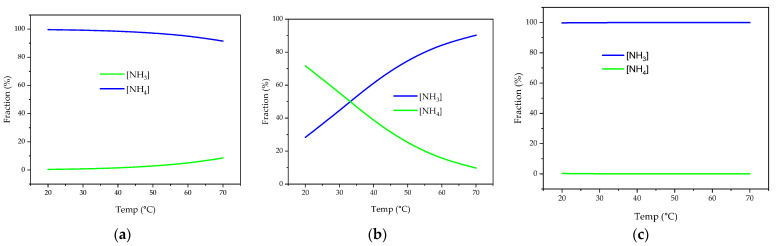
Effect of temperature on NH_3_/NH_4_^+^ speciation at (**a**) pH =7, (**b**) pH =9 and (**c**) pH = 12.

The separation mechanisms of nitrogen compounds with NF and RO include Dannon potential, steric effects, membrane potential, and size exclusion [123]. Considering these mechanisms, the molecular charge and structure are important for nitrogen rejection with NF and RO. The common forms of nitrogen in wastewater are urea, ammonium ion, ammonia, nitrate, and nitrite. The dominance of one form over the other depends mainly on pH and biological activities. As mentioned in the previous section, urea is converted to ammonium enzymatically, whereas ammonium is converted to ammonia, nitrate and nitrite by the nitrification/denitrification processes in an alkaline environment. Lee and Lueptow [124] studied the rejection of the nitrogen compounds with NF, RO, and low-pressure RO theoretically and experimentally. They found that Dannon potential and the ratio of the solute radius/pore radius (steric effect) are the dominant rejection mechanisms for nitrogen compounds in NF and RO. The transport of nitrogen compounds through NF and RO was mainly governed by diffusion, and convection and electromigration only played small roles, especially for charged molecules. Convection had about 20% and 30% contribution to uncharged nitrogen (urea) compounds transport through RO and NF membranes, respectively. The small size and charge neutrality of urea makes rejecting it with high levels hard even with the RO membrane. Nitrate and nitrite have the same rejection as ammonium with RO membranes, but with NF their rejection is affected by the counterions. The rejection is higher if the counter ions were multivalent than if they were monovalent. This highlights the importance of using natural samples with their complex environment in testing membranes for nitrogen recovery. Testing synthetic solutions may result in misleading conclusions about the membrane rejection capacity. Since the nitrogen rejection is greatly influenced by the electrochemistry of the solution and membrane, special attention should be paid to the pH of the solution. The charge of the membrane surface is believed to stem from the dissociation of the ionic groups which exist on it [125]. These ionic groups can be of acidic, basic, or amphoteric nature depending on the membrane material [123]. For instance, the solution pH change of polyamide RO membrane has been seen to alter the surface charge and consequently affected ammonium rejection [29]. At pH > 6, the carboxylic and mine groups on the membrane surface get deprotonated giving rise to a negative charge. This leads to rejection of negative ions through repulsion and as a result, positive ions such as NH_4_^+^ are rejected to maintain electroneutrality [126]. At pH< 6, carboxylic and amine groups get protonated and making the membrane surface charge neutral which allows the passage of monovalent ions. To maximize nitrogen rejection with NF and RO filtration, converting it to a charged molecule and maintaining pH levels that prevent electromigration are essential. Temperature should also be considered as it affects ammonium speciation (see Figure 9) and organic carbon volatility in addition to its effects on reaction kinetics.

Polymeric or ceramic membranes can be used for nitrogen recovery. Ceramic membranes have attractive traits when considering nitrogen recoveries, such as ease of cleaning, narrow pore size distribution, wide pH range, and long operating life [127]. However, their high cost gives polymeric membranes a competitive edge over them. Given the low quality of wastewater, frequent cleaning and replacement may be required for maintaining stable operation. Hence, using low-cost polymeric membranes makes more economic sense. Zarebska et al. [127] collated literature data for the polymeric membranes used for nitrogen recovery from wastewater and compared them in terms of fouling tendency using surface energy, contact angle, and work of adhesion as criteria. The membrane materials used in pressure-driven filtration for nitrogen recovery include polypropylene (PP), polyvinylidene fluoride (PVDF) and polytetrafluoroethylene (PTFE), polyamide (PA), polyethersulfone (PES) and polysulfone (PSU). The fouling tendency followed the following order PTFE > PP > PES > PSU > PVDF > PA, which correlated well with surface energy and contact angle. The higher the contact angle and the lower the surface energy, the higher the fouling propensity. The work of adhesion can be used as an indicator of the ease of cleaning. The higher the work of adhesion, the harder is to remove foulants. The work of adhesion of polymeric membrane was calculated to be in the order of PA > PSU > PVDF > PES > PTFE > PP.

A summary of nitrogen recovery studies with different pressure-driven membranes is presented in Table 3. The summarized literature tables presented in this section and the following sections are intended to present studies that cover the most important aspects of membrane applications, such as tested feed types, membrane materials, and operational conditions. The flux is expressed in L/m^2^·h, henceforth abbreviated as LMH. One obvious point that can be noted is that pressure-driven membranes were mostly used with animal wastewater. Also, the most used membrane material in RO technology is PA and this is due to its high selectivity and wide pH operational range [127]. MF, UF, and even NF achieved low to moderate nitrogen rejection and this is expected due to the poor separation capacity of these membranes. RO has the highest NH_4_^+^ rejection, but the flux is much lower than other pressure-driven membranes. High nitrogen rejection was reported in some MF, UF, and NF studies, but this is believed to be due to nitrogen losses unaccounted for. The most challenging issue with pressure-driven membranes is the high energy requirement and rapid development of fouling, especially for high solids streams such as animal and food waste. Fouling occurs in all membrane types, but it is the most recalcitrant in pressure-driven membranes due to the application of external pressure that drives and deepens the adhesion of contaminants onto membrane surfaces and within its pores. Fouling is a complex process affected by a large number of intertwined factors, such as solution physical and chemical characteristics, the nature of foulants, membrane properties, and operating conditions [128]. Parallel to this, several measures are normally implemented to combat this problem, such as tuning the hydrodynamic conditions, modifying membrane properties, altering feed properties, and applying effective pre-treatment techniques [129,130,131]. For relatively low to moderate solids streams, the pre-treatment can reasonably be simple and energy-efficient. A recent study reported an interesting approach to using the concept of dynamic filtration as a cost-effective technique for reducing solids and other pollutants in the primary effluent [36]. This study proposed the use of flat sheets of monofilament polyamide to serve as a substrate for the self-formed dynamic membrane. This kind of barrier can improve the effluent quality to be used as feed for nitrogen recovery with a pressure-driven membrane. This process takes time to build a stable layer of active biosolids. However, coagulants can be added to expedite the process. Regardless of pre-treatment efficiency, periodic physical and chemical cleaning are usually applied as standardized industry practices for maintaining membrane operation.

## 5. Thermally-Driven Membrane Processes

Membrane distillation (MD) is a thermal-based membrane separation process driven by a vapor pressure gradient caused by a temperature difference across microporous hydrophobic membranes [144]. It keeps the non-volatile compounds and liquid water in the streams while allowing the volatile or gaseous substances (e.g., NH_3_) to pass through the membrane. Therefore, the product achieved is theoretically 100% free from solids or non-volatile substances [145]. MD has been used in many applications, such as seawater desalination, the food industry, the treatment of industrial wastewater, and resource recovery from various liquid streams. The past 10–15 years have witnessed a growing interest in the utilization of MD technology for ammonia recovery [146,147,148]. Different MD configurations have been used to recover ammonia from waste streams including, direct contact MD (DCMD), Vacuum MD (VMD), Sweep gas MD (SGMD), and air-gap MD (AGMD) [149]. For the first and last configurations, absorption solutions, such as inorganic acids are used for direct scavenging of the diffused ammonia through the membrane. For the second and third configurations, the ammonia gas stream is extracted using vacuum pumps and sweeping gas (e.g., air) and bubbled through absorption solutions.

A microporous hydrophobic membrane material is required, and different polymeric materials have been used for MD. These include PVDF, PP, and PTFE. Among them, PTFE is the most promising material due to its high chemical stability and the lowest surface energy. Thus, the risk of membrane wetting is low. During the MD process, the partial pressure of volatile organic matter and ammonia in the liquid is higher than the water partial pressure and would be better transported across the membrane. Most of the non-volatile substances such as phosphorus are retained and concentrated in the feed side. As compared to other conventional ammonia recovery including chemical precipitation and ammonia stripping, chemical post-treatment is not required for the product. However, this depends on the quality of the feed. If hazardous volatile carbon such as poly aromatic hydrocarbon (PAH) or other micro-pollutants, then removal of these compounds would be required to obtain a high-quality recovered product.

As compared to pressure-driven membrane processes, MD has a lower operating pressure and susceptibility to membrane fouling and can handle feed streams with lower quality [150,151]. One of the attractive characteristics of MD is the possibility of utilizing low-quality thermal energy such as geothermal and energy by-products from wastewater processes such as heat and biogas [152,153]. Many research studies have demonstrated the potential of using DCMD and VMD modules for recovering ammonia from various waste streams. However, very few studies have investigated the potential of AGMD and SGMD for nitrogen recovery, and this is apparent in Table 4 which sums up the outcomes of MD studies. There are some useful trials that compared these different technologies for the same treatment conditions. The outcomes of these studies might have the answer to why some MD configurations were more common for nitrogen recovery compared to others. Ding et al. [154] determined experimentally the ammonia mass transfer coefficient and selectivity for VMD, DCMD, and SGMD under the same operating conditions. They found that the mass transfer coefficient followed an order of VMD > DCMD > SGMD, while the order of the selectivity was DCMD > SGMD > VMD. Another study explored water recovery and ammonia rejection with AGMD, DCMD, and VMD using black water as a feed stream [155]. It was found that AGMD exhibited the most stable rejection of ammonia with different operating temperatures, and it was directly related to solution pH. VMD achieved the highest ammonia rejection, while DCMD rejection was low at temperatures < 40 °C. In most pH and temperature ranges applied, AGMD had rejection efficiency higher than DCMD and lower than VMD. DCMD is also known to suffer from low thermal efficiency due to heat loss by conduction. The outcomes of these two studies suggest that VMD is probably the best MD configuration for nitrogen recovery. Although, more studies on AGMD and SGMD are required to confirm this conclusion.

Many factors affect the efficiency of nitrogen recovery with MD. Among them, pH and temperature are the most studied and probably the most important ones. This emanates from the fact that nitrogen is recovered in gaseous form in MD technology. The speciation of ammonia and ammonium for the common temperature and pH ranges have been calculated using Equations (1) and (2), and the results are presented in Figure 9. In a highly alkaline environment (pH = 12), the temperature does not affect ammonia production as all the ammonium is converted to ammonia. In this case, MD turns into GPM (Section 9). At neutral pH, temperature increase has only a small effect on ammonia production. In comparison, at a pH close to the pKa of ammonia, the temperature has a significant impact on ammonia production. Luckily, most waste streams are at a pH level close to the pKa of ammonia (Table 2), which makes MD suitable technology for nitrogen recovery. Higher feed temperature creates advantageous conditions for ammonia recovery: (1) higher partial pressure of ammonia vapor (2) lower ammonia solubility in aqueous solution (3) lower liquid viscosity. However, applying high temperature is costly, and it can exacerbate the dilution issue of the recovered nitrogen as more water vapor transfers along with ammonia gas. Temperature rise can be compensated by raising the pH of the solution. Depending on the available resources, either temperature or pH can be adjusted to achieve high nitrogen recovery. If waste heat is available, then chemical addition can be set to a minimum and vice versa.

Despite the high nitrogen recovery achieved with MD technologies (Table 4), these technologies are still an unfavored option for nitrogen recovery due to high energy demands and fouling and wetting issues. The common foulants occurring in MD, especially for nitrogen recovery applications, are inorganic salts. The elevated temperature can weaken microbes and leads to the volatilization of carbon, hence organic and biofouling occurrence in MD are less compared to inorganic fouling. Inorganic constituents can act as a bridge for anchoring organic foulants [156]. Inorganic foulant accumulation can be alleviated by dropping the feed pH to increase the solubility of scaling salts [157]. However, in this case, nitrogen is not recovered in a high-quality product, rather it is concentrated in the feed stream. This scenario can be feasible for some streams that are rich with minerals such as urine and landfill leachate where NH_4_^+^ can be concentrated for later recovery through struvite precipitation using existing phosphorous and manganese or supplementing it externally. Raising pH for other streams is probably more feasible due to the low product value of the concentrated steam compared to the high-quality ammonium salts produced from NH_3_ gas recovery. If water recovery is of interest, then acidifying the feed is logical.

## 6. Osmotically-Driven Membrane Processes

The applications of osmotically driven membranes for nitrogen recovery are represented by the utilization of FO membrane technology in its two operational modes FO and pressure retarded osmosis (PRO) [172]. In FO mode, the active layer is set to face the feed solution, while in the PRO mode, the active layer is facing the draw solution [173]. The structure of the FO membrane comprises an active smooth layer and a porous support layer. Both symmetric and asymmetric membranes have been reported to be used for nutrient recovery [172]. However, the application of the asymmetric FO membrane has been more common in the literature [173]. Concentration polarization (CP) is a phenomenon that occurs in all membrane technologies. External concentration polarization (ECP) is common in all membrane technologies including FO and it entails the rise in solute concentration in the liquid layer adjacent to the membrane’s active surface. ECP hinders mass transfer across the membrane by reducing the concentration difference between each side of the membrane selective layer. Internal concentration polarization (ICP) is a problem specific to FO membrane, where draw solute ions enter the porous support layer. In FO mode, a symmetric membrane experiences only ECP, whereas an asymmetric membrane experiences both ICP and ECP [174]. Although PRO mode increases permeate flux and decrease CP, FO mode is preferred due to the easiness of fouling removal from the active layer compared to the porous support layer [173]. ECP can be alleviated by changing the flow conditions to create turbulences close to the active surface of the membrane [175]. ICP can be mitigated by selecting suitable design parameters for the membrane. The salt diffuses through the support layer in the most common operational mode, FO. Hence, selecting the proper structural parameters of the support layer such as thickness, porosity and tortuosity can reduce ICP effects on the process [176]. It was found that the smaller the structural parameter (*S*), the lower the ICP [177]. *S* is defined as the effective solute diffusion path in a porous media. *S* is expressed in membrane structural parameters as *τl/ε*, where *τ* is the tortuosity, *l* is the thickness and *ε* is the porosity of the support later [178].

Reverse salt flux (RSF) is an intrinsic challenge of FO technology. This phenomenon is defined as the diffusion of the salt through the FO membrane from the draw solution to the feed. This phenomenon causes four problems: (i) dilution of draw solution, which requires replenishment or concentration (both of which incur a cost), (ii) may alter the feed solution chemistry and biological environment (iii) causes loss in the osmotic driving force resulting in lower water flux and (iv) induces ICP [179,180]. The diffusion of the salt to the feed side can instigate scaling and negatively impact the sludge structure in wastewater processes [181]. Some studies found that rising salts levels in biological sludge can lead to microbial inactivation, loss of mixed liquor suspended solids (MLSS), reduction in flocs size, and increase in retention time [182,183,184]. Elevated levels of salts can induce the secretion of organic cellular materials that accompany the endogenous respiratory stress response of microbes to such environment [185]. These materials can exacerbate biofouling and make it hard to remove due to the forming of a protective layer over the adhered microbes [186]. Membranes with high perm-selectivity (permeability/selectivity) should be chosen for reducing RSF [187].

What sets FO apart from pressure-driven membranes in wastewater applications is low energy demands and ease of fouling removal (development of irreversible fouling) [188]. Additionally, FO utilizes nonporous membranes that unlike the porous membranes used in MF and UF can reject soluble organic matter, nutrients, and dissolved heavy metals and micropollutants [176]. However, FO is still not immune to fouling. The major fouling categories that have been reported in the FO process for nutrient recovery are biological [189], inorganic, and particulates [190]. Fouling accumulation on the membrane surface can negatively impact the process performance through the addition of hydraulic and osmotic (foulants-enhanced CP) resistances [13]. Depending on the fouling types, three strategies have been reported to alleviate this problem: (1) feed pre-treatment such as using disinfection for controlling biofouling [189] or removal techniques for controlling particulate, organic, and inorganic fouling [191], (2) applying hydrodynamic forces through changing flow conditions, creating pulse flow or using spacers specifically designed for this purpose [192], (3) using ultrasonic vibration [190] and (4) designing or modifying the membrane surface with high fouling resistance [193,194,195,196]. Given the high organic content of waste streams, disinfecting them is not an efficient strategy. Large amounts of disinfectants are required in this case due to the scavenging of disinfectant by organic carbon forming hazardous disinfection by-products. Some membranes’ materials are more foulant resistant than others. The touchstones for an ideal FO membrane are high water flux and solute retention, low fouling and CP propensity, and high mechanical and chemical resistance [172]. Regardless of the membrane nature and the flow conditions applied, a pre-treatment for the feed is required especially when waste streams with high foulants concentrations are utilized (see Table 2). Normally, solids and colloidal separation is applied for pre-treating the feed solution to reduce fouling formation. Membrane cleaning is also required as part of the maintenance routine. Both pre-treatment and membrane cleaning can increase the cost of nitrogen recovery with FO. So, they need to be carefully selected to suit the characteristics of the feed solution and overall conditions of the treatment environment.

The FO process is affected by several factors such as temperature, flow rate, pH and composition of feed and draw solution, and the nature of the membrane material [172]. Among the aforementioned factor, the most influencing ones are the type of FO membrane and the used draw solution [197]. There is an array of membrane materials that have been applied for nitrogen recovery. These materials include cellulose triacetate (CTA), thin film composite (TFC), aquaporin-embedded membranes, and virgin and surface-modified polyamide TFC membranes [172,198]. Despite the higher surface smoothness, hydrophilic nature, and neutral surface charge of TFC, they were found to have a higher fouling tendency compared to CTA [199]. However, CTA is prone to biodegradability which could lead to loss of membrane rejection and failure in the process [200]. Table 5 shows a summary of the outcomes of recent FO studies for nitrogen recovery using different membrane materials and draw solution options. Surface modification of the FO membrane can improve water flux and nitrogen rejection. It was found that grafting polyamide membranes with ethylenediamine (EDA) and 2-aminoethanol (AEA) improved ammonium rejection even at elevated pH [201]. The ammonium ion rejection improvement was attributed to the increase in the carboxylic group density with grafting that in turn alleviated cation exchange between NH_4_^+^ and Na^+^. Although this research area is of great interest, it is outside the scope of this work. Readers are referred to a review work on this topic reported by Xu et al. [197]. There are other membrane modification practices, such as the incorporation of aquaporin into the structure, which have been shown to improve membrane antifouling properties [198,202].

There is a range of draw solutions that have been utilized for nitrogen recovery using the FO process. Johnson et al. [203] listed several criteria that should be available in an effective draw solution. These are (i) ability to generate high osmotic pressure, (ii) low viscosity that allows easy pumping and recirculation, (iii) low reverse solute flux, (iv) high diffusion coefficient to reduce the impact of ICP on the process, (v) availability in large quantities, (vi) affordability (vii) easily regenerated at low cost and (viii) impose no environmental or health risks on the finished product. Johnson and co-workers highlighted that the availability of the aforementioned criteria in a single draw solution might be hard to find, and quoted the example of NaCl with low ICP tendency but high RSF. NaCl and MgCl_2_ seem to be the most common draw solutions used for a nitrogen recovery application. The selection of MgCl_2_ draw solution has been motivated by many factors among them the exploitation of RSF of Mg for struvite precipitation on the feed side [204]. Mg^+2^ has a hydration radius larger than the commonly used cation Na^+^, which reduces RSF [122]. Multivalent salts offer higher osmotic pressure as opposed to monovalent salts for the same concentration due to the production of a larger number of ionic species upon dissociation [205]. However, divalent ions were found to promote organic fouling through their interaction with polysaccharides [206]. The selection of inorganic draw solutions for various FO applications was carefully analyzed by Achilli et al. [122]. Another class of draw solution that has been tested for nitrogen recovery is commercial fertilizers. The advantage of these materials is that they can be used in their diluted form for agricultural purposes without the need for regeneration [203]. Ionic organic compounds such as ethylenediamine tetraacetic acid disodium [207] and sodium acetate [208] have also been utilized as draw solutions. In addition to the abovementioned draw solutions, there are more options which have been thoroughly discussed by Johnson et al. [203], though the focus of this work was on water recovery.

The decrease in draw solution salinity is managed normally either through using a larger quantity compared to feed solution [209], applying systematic salt dosing [210], or re-concentration using RO or MD [172]. The first two approaches can only be applied to small-scale or batch processes. Hence, only re-concentrating with RO and FO can be applied for large-scale applications. These two membrane solutions are costly but can be attractive when another waste or saline stream is used as a draw solution (e.g., RO brine or seawater). In this case, there is an added incentive represented by the pure water recovery. This synergy can be realized if the sources of the feed and draw solutions are in geographical proximity.

**Figure 10:**
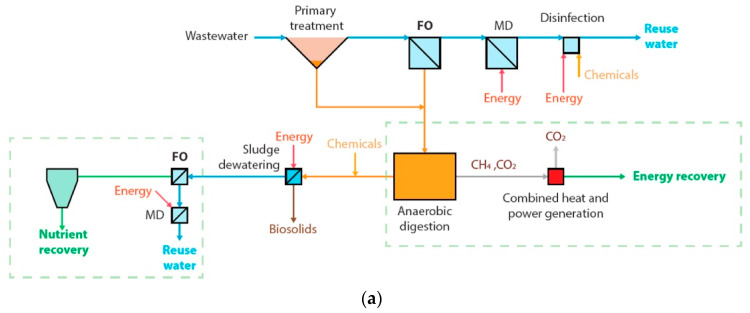

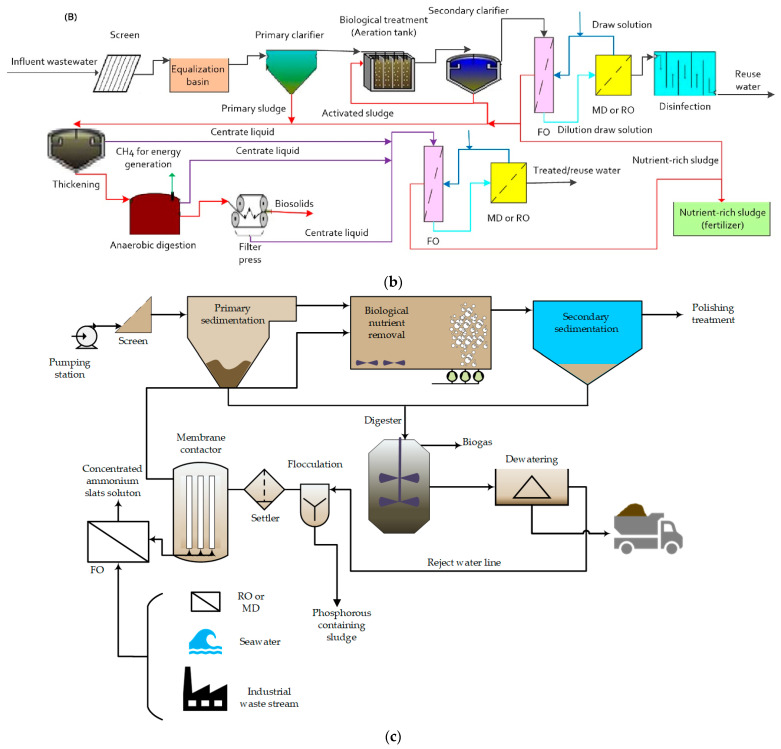
Conceptual large-scale design for FO application for nitrogen recovery from domestic wastewater treatment plant proposed by (**a**) Reprinted from Ansari et al. [13], copyright (2015) with permission from Elsevier, (**b**) Reprinted from Jafarinejad [172], copyright (2021) with permission from Elsevier and (**c**) this study.

Conceptual designs for large-scale applications of FO for nutrient recovery from wastewater have been proposed by Nguyen et al. [211], Ansari et al. [13], and most recently Jafarinejad [172] (Figure 10). The illustration for Nguyen et al. [211] design has not been included in the figure. It entails the application of FO in two stages within the wastewater treatment train for recovering nutrients from the effluents of biological treatment and the digester. Although the designs proposed in the literature reflect the forward-thinking of the researchers, they overlooked some important aspects, such as the need for pretreatment for the FO feed as the supernatant of clarifier or the centrate of the digester contains high levels of solids, organic and inorganic constituents that can foul FO in a short time. The other problem is the low water flux of FO and, considering the large volume normally treated in wastewater treatment plants, this may require an FO unit with a huge footprint that could render the process costly in terms of capital investment and operation. With these designs, the concentrated feed does not only have nutrients, it contains also all other constituents such as pharmaceuticals, endocrine agents, pathogens, etc. Hence, for a more practical approach, we propose the use of GPM with FO for nitrogen recovery from the reject water stream only as the volume of this stream is manageable (Figure 9c). It should be noted that this configuration is currently being investigated by the NPHarvest team at Aalto University in Finland who developed an efficient membrane contactor for nitrogen recovery from streams with high solids content [10,23,26]. The team also devised a cost-efficient ballasted flocculation-sedimentation process for phosphorus and solids removal. The advantage of this system is producing high-purity ammonium salts and phosphorous-containing sludge with a low concentration of pathogens and micropollutants. The returned reject water has a lower nitrogen concentration that can lower aeration energy requirements for the biological treatment process. Though this depends on the targeted nitrogen removal percentage and the volume of the reject stream. The available options for the draw solution regeneration are similar for all the designs in Figure 10. The draw solution source and regeneration are important factors that could significantly impact the economic feasibility of FO applications.

## 7. Biologically-Enhanced Membrane (BES) Processes

The biologically enhanced membrane (BES) systems for nitrogen recovery encompass three main technologies: anaerobic MBR (AnMBR), osmotic MBR (OMBR), and photobioreactor membranes. Different configurations and designs have been reported for these technologies. It should be mentioned here that the bio-electro-chemical membrane process could be counted as part of the BES family, but we chose to address this branch of membrane technologies in Section 8.

### 7.1. AnMBR

The rationale behind the development of AnMBR is the combination of membrane technology (mostly MF or UF) with the conventional activated sludge (CAS) process [12,226]. In comparison to CAS, MBR has the advantage of better effluent quality, compactness, and easier operation and management [227]. Despite these advantages, MBR technology still converts reactive nitrogen into N_2_ and releases it into the atmosphere. This does not promote circular economy principles and leads to environmental problems. AnMBR has emerged as an alternative MBR configuration that improves the management of carbon and nutrients in the MBR process. AnMBR was first introduced for treating high-strength wastewater [228]. The growing interest in AnMBR has mainly been driven by the capacity of this system to convert carbon to methane-rich biogas. The produced biogas can offset part of the spent energy for operating the system [229]. Not requiring aeration is another distinctive feature that set AnMBR apart from MBR and CAS. Nutrients can also be converted into reactive forms with AnMBR [12], which can be recovered in subsequent processes or used directly if the quality of the effluent is acceptable.

The detailed biological process or the AnMBR is outside the scope of this work, but a brief description is provided here. The anaerobic digestion of waste in the AnMBR involves four stages: hydrolysis, acidogenesis, acetogenesis, and methanogenesis [12]. These stages are performed by the harmonious work of different groups of microbes, namely fermentative bacteria, syntrophic acetogens, homoacetogens, hydrogenotrophic methanogens, and aceticlastic methanogens [230]. Among these microbial groups, methanogen is the most important one due to its role in converting the decomposed carbon produced from the first three stages into methane [12]. Protecting this group from being washed out is one of the important functions of the membrane in the AnMBR system. The integration of the membrane with the anaerobic process can be done in three configurations: side-stream, submerged or housed in an external chamber [12].

Ammonia is produced from the biodegradation of organic nitrogen (e.g., protein) by anaerobic microbes [231] and passes through the membrane (MF or UF) to the effluent. The produced ammonia by AnMBR needs to be recovered by separation or concentration. To this end, a number of techniques have been suggested such as MD [171], electrolysis [232], and photobioreactor [233]. A pre-concentration of wastewater may also be required for maintaining effective biological processes in AnMBR. It has been reported that wastewater with a COD content of >1000 mg/L is necessary for achieving high levels of biogas production and nutrient removal [234]. This is not the only challenge associated with the AnMBR application. The operating environment for AnMBR is either mesophilic (30–40 °C) or thermophilic (40–50 °C) [235], and this restricts its use in cold seasons and places unless external thermal energy is used. Like any biological process, the presence of inhibitory substances in the waste stream can significantly deteriorate AnMBR performance, if not completely stop, the activity. Salinity has been reported to negatively impact anaerobic processes and leads to the exacerbation of membrane fouling [231,236]. The accumulation of ammonia (which is a product of this process) to a level of >3.5 g/L can be toxic to anaerobic microbes. Ammonia toxicity to anaerobic microbes emanates from the inhibition of cytosolic enzymes and the increase of pH and cations [12]. High sulfate can also harm anaerobic processes. Sulfate-reducing bacteria can compete with methanogens over the available carbon [230]. Sulfate can promote the precipitation of the methanogen micro-nutrients and the production of hydrogen sulfide, a toxic corrosive gas [237]. Membrane fouling is another concerning issue for AnMBR. The common foulants experienced in this system are microbial cells and debris. Normally, biogas is sparged for fouling control, but sometimes vigorous sparging is required which heightens the energy demands for AnMBR to exceed that of MBR [238]. This is expected as AnMBR runs with high hydraulic and sludge retention times (HRT and SRT) that result in severe membrane fouling [239]. Song et al. [12] proposed a list of strategies to overcome these challenges that can help to improve the stability and productivity of AnMBR, but they may increase the process cost.

### 7.2. OMBR

**Figure 11:**
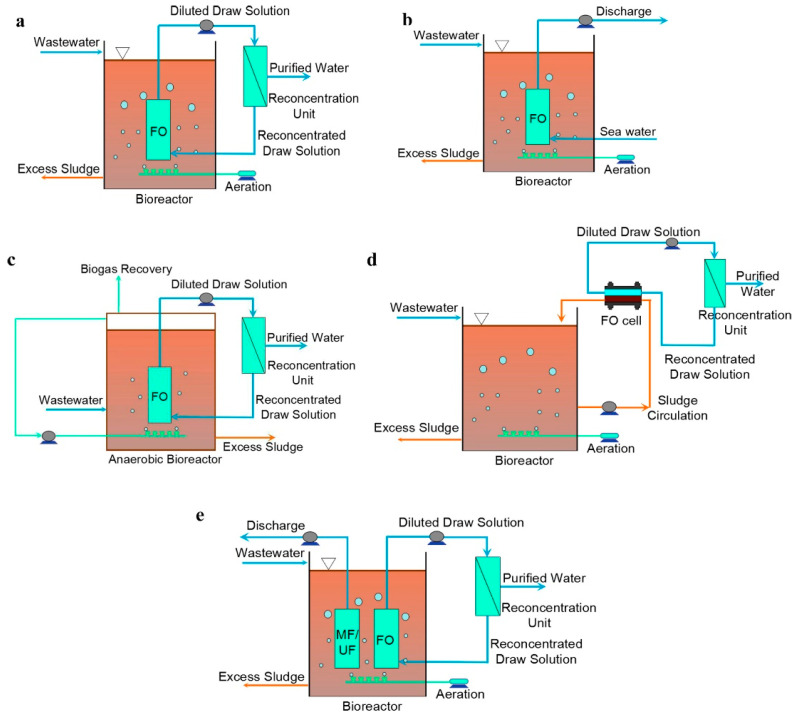
OMBR configurations: (**a**) submerged aerobic OMBR with draw solution regeneration unit, (**b**) submerged aerobic OMBR with open loop draw solution, (**c**) side-stream anaerobic OMBR for biogas production, (**d**) side stream aerobic OMBR with draw solution regeneration unit and (**e**) aerobic OMBR with draw solution regeneration unit and salt leak membrane system (MF/UF). Reprinted from Wang et al. [176], copyright (2016) with permission from Elsevier.

OMBR consists of a bioreactor, FO separation unit, and draw solution replenishment/regeneration system. The two distinctive differences between OMBR and AnMBR are the use of a nonporous FO membrane for separation and aeration for maintaining effective biological activities and controlling membrane fouling in the former, while porous MF or UF are used for separation and biogas recirculation is utilized for fouling control in the latter [176]. OMBR exists in different configurations depending mainly on the purpose of the application and the way the draw solution is managed as seen in Figure 11 [176]. The first two configurations (a and b) are the standard and most common ones. Configuration a is more energy intensive, but it produces clean water with the aid of RO or MD. Configuration b is applied when there is a readily accessible source for draw solutions (e.g., seawater or industrial stream). Configuration c is the only one with an anaerobic operational environment, and it is normally applied for producing biogas. As mentioned in Section 7.1 anaerobic conditions requires high-concentration wastewater. FO rejection of organics and nutrients can enrich the feed side and reduces the need for a pre-concentration step [240]. In the side stream configuration, the FO unit is set outside the biological basin. The design has the disadvantages of a high fouling tendency due to the high solids of the mixed liquor and narrow flow channels in FO [241], high energy requirement for pumping, and the breakage of sludge flocs that can deteriorate the biological activities [242]. Hence, this configuration has not gained popularity for wastewater treatment and resource recovery. The last configuration involves the integration of MF or UF membrane for mitigating salinity accumulation, known as the *salt leak*. The discharge of the porous membrane can be recirculated back to the influent line of wastewater. The withdrawn salt constituents might contribute to phosphorous removal that is normally conducted in wastewater treatment plants using iron salts.

OMBR is prone to the same problems mentioned in Section 6. The strategies suggested for mitigating these problems there are also applicable to OMBR. The unique challenge in OMBR is the conflicting effects of salt accumulation on the performance of the system for nitrogen recovery. On one hand, the accumulation of salts can slow down the oxidation of ammonia to nitrate under aerobic conditions leading to enriching the feed with ammonia [243]. On the other hand, salt accumulation can negatively affect the activities of the aerobic microbes leading to the deterioration of the quality of the OMBR effluent.

NH_4_^+^ rejection with different configurations of OMBR varies between 55% and 98% as shown in the reviewed studies in Table 6. Different designs of OMBR systems were used in the literature with submerged FO with plate and frame configuration being the most popular. Even though the side stream configuration had the highest ammonium rejection, it is not feasible in the long run due to fouling issues and structural damage of sludge flocs. In some studies, almost no rejection of NH_4_^+^ was reported with OMBR due to the effective aerobic biological activities that converted it to nitrate [244,245]. Recently, innovative biologically-based hybrid systems have been proposed and tested for further improvement of anaerobic OMBR systems such as the integration of the moving bed concept and the combination with an up-flow anaerobic sludge blanket [246,247]. OMBR seems to be an effective technology for not only enriching wastewater with nutrients but also can be utilized for water recovery when combined with suitable membrane technology.

### 7.3. Photobioreactor Membranes (PBRMs)

This type of membrane system harnesses the ability of phototrophic organisms, such as microalgae, to convert carbon dioxide and nutrients to biomass that can be harvested with aid of membrane separation. The integration of membranes with photobioreactors (PBRs) has emerged as an effective solution for the poor settlement of microalgae [254]. Another attractive trait of PBRMs is their smaller footprint compared to conventional PBRs and open ponds [255]. PBRMs have the capacity of fine-tuning HRT and SRT, which is needed for the efficient operation of photobioreactors [256]. In this system, nitrogen is recovered in the form of biomass that can be used in the production of different valuable materials such as pharmaceuticals, biofuels, and animal food [257]. It was suggested that a nitrogen/phosphorus ratio of 5–30 is required for the successful growth of microalgae [258]. Such a ratio is available in domestic and agricultural wastewater with nitrogen and phosphorous concentration ranges of 15–90 mg/L and 4–20 mg/L, respectively [259]. The preferred form of nitrogen for microalgae is ammoniacal nitrogen which can be converted directly to amino acids [260]. However, the assimilation of nitrate or nitrite may require several cycles of reduction [261]. Nitrogen uptake can negatively be affected by the phosphorous deficiency. This means that PBRMs are not feasible to be applied in streams like reject water where most of the phosphorous are already removed in the proceeding steps.

A typical PBRM system consists of a membrane submerged in a photobioreactor supplied with light and CO_2_ sources. Since fouling and biomass accumulation on the membrane surface takes place frequently in this system, aeration is normally applied [261]. MF and UF are commonly used in the PBRM system, however, some studies have utilized nonporous FO membrane (the system is referred to as OMPBR) [262]. Natural light is the most feasible light source for this technology, however, the seasonal and diurnal fluctuations as well as the unavailability at night times make it unreliable source for maintaining a consistent operation. Hence, mostly artificial light sources are used for operating PBRMs.

PBRMs offer attractive solutions for today’s world environmental challenges such as the utilization of CO_2_ for producing value-added products and removing nutrients from wastewater in a sustainable environmental way. To get maximum benefits from these systems, the right operational conditions need to be applied along with the selection of suitable waste streams and algal strains. The selection of appropriate HRT, SRT, and HRT/SRT is an important factor for microalgae biomass growth and nutrient uptake. High HRT is required for effective nutrient removal, but high SRT negatively affects microalgae growth and induces severe membrane fouling [261]. For the balanced operation of PBRMs, most studies select moderate HRT and SRT [263]. A temperature higher than 25 °C was also reported to have a negative effect on microalgae growth [261]. Reactor design plays an important role in achieving high photosynthetic efficiency. Designing a reactor with a shorter light path was found to improve microalgae growth [264]. Choosing microalgae strains that can tolerate the fluctuation in wastewater quality is vital for maintaining a stable process. Some studies used a mixture of microalgae and wastewater microbes for nitrogen removal. For instance, Amini et al. [265] achieved 94% NH_4_^+^ removal using an inoculum ratio of 5:1 of microalgae to waste-activated sludge. Selecting membrane technology that suits the available energy resources is important for the economic feasibility of the process. If there is a water source or waste stream with high osmotic pressure, FO can be utilized in lieu of MF or UF. A comparison study between PBRM using MF and OMPBR found that the latter had better nitrogen removal [266].

In a recent review of PBRM application for nutrient recovery, it was reported that this technology could achieve nitrogen removal of 30–100% [261]. However, the collated results in this study were gathered from investigations that used synthetic wastewater or real wastewater in a controlled environment for short tests. Several challenges face the full-scale application of PBRMs for nutrient recovery such as the adaptability of microalgae to changes in water quality and environment, the complex physical-biological process that is hard to optimize, irreversible fouling caused by external algal organic matter, maintenance of certain microbial diversity throughout the process and instability of the system for long term operations [261,267]. PBRMs can be a promising nutrient recovery technology for content defined and stable streams, but not for common waste streams that are known by the dynamicity of their quality.

## 8. Electro-Chemical Membrane Processes (ECMs)

**Figure 12:**
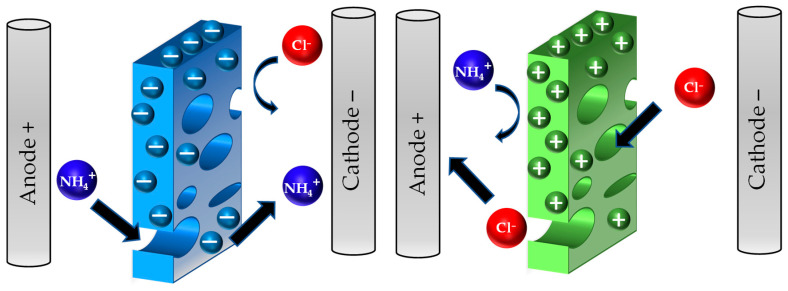
Simplified illustration of an electrochemical membrane system for nitrogen recovery.

One of the recent advances reported in membrane separation science is the electrochemical membrane processes. Electrochemical technologies integrated with membrane filtration have been routinely suggested to allow remediation treatment and diminish the limitations of the standalone membrane process [268]. Lately, membrane-based electrochemical processes have witnessed a distinct interest as prospective technologies for treatment and nutrient recovery from wastewater. Membrane capacitive deionization, electrodialysis membrane, and electrochemical filtration systems are the major examples of selective electrochemical membranes. These processes have easily found their way to versatile applications, e.g., desalination, energy production and resources recovery from waste streams, and wastewater disinfection [269]. Typically, an electrochemical system comprises a semi-permeable ion exchange membrane placed in between the cathode and anode (Figure 12). For instance, electrodialysis (ED) harnesses ion-exchange membranes beside an electrical potential as a driving force. ED is a membrane-based separation technique where anion exchange membranes and cation exchange membranes are arranged alternatively [270]. Here, the ion-exchange membrane carries charged functional groups and could be either homogenous or heterogeneous depending on the way the functional group is attached to the membrane. Meanwhile, the backbone endows the essential dimensional stability and strength of the membrane. Depending on the charged group associated with the monopolar ion exchange membrane it could be either an anion-exchange or cation exchange membrane [271]. Membrane material selection is crucial to determine ionic properties bestowed upon the system performance [269]. Likewise, membrane capacitive deionization (MCDI) is a modified form of the classical capacitive deionization (CDI) process where membranes are integrated into the system. For ion removal, the MCDI technique harnesses an electrical potential gradient across an aqueous solution that inflows in between oppositely placed porous electrodes, where ion-exchange membranes are positioned in front [272].

The applications of electrochemical membranes can extend to energy generation from organic pollutants found in wastewater or so-called microbial fuel cells (MFC) [269]. Another bio-based electrochemical membrane system is the microbial electrolysis cell (MEC) [257]. In these systems, chemical energy is converted to electrical energy through a series of microbially catalyzed reactions at the anode chamber [273]. Organic carbon is oxidized by heterotrophic microbes releasing electrons that transfer through a resistor to the cathode where there is electron acceptor species such as air are present [257]. The thermodynamic reaction favorability is what distinguishes these systems from one another. The anode reaction in the MFC is thermodynamically favorable, so electrical energy can be recovered without the need for external energy input. On the contrary, the cathode reaction in the MEC is not thermodynamically favorable and this requires the use of external energy to drive the ions transfer in the cell [274]. The oxygen reduction at the cathode generates hydroxyl ions and this increases the pH of the catholyte at the cathode surface [275]. Although pH increase is localized, it can still contribute to ammonium/ammonia conversion and consequent nitrogen recovery. In quite recent studies, MFCs have been repeatedly reported in the literature for nutrient concentration and recovering water and energy from wastewater [276,277,278]. Sustainable bio-electrochemical treatment of nitrogenous mariculture wastewater was reported by Jiaqi et al. [279]. The study merged the synergy of cathodic photo electro-catalysis and algae for promoting nitrogen removal, where 77.35% inorganic nitrogen and 94.05% NH_4_^+^ were obtained. Enhancing resource recovery from wastewater streams was also carried out via an electrochemical-osmotic system using nanofiltration membranes. Instead of applying FO membranes, recent work conducted by Wang et al. [280], harnessed polyelectrolyte multilayer nanofiltration membranes for electricity generation, enhanced water production, and metal recovery from wastewater. In their work, the nanofiltration membrane was synthesized through two oppositely charged polymers deposited alternately. The authors disclosed a novel avenue to promote a high-performance electrochemical-osmotic system for reclaiming multiple resources from wastewater.

With the ongoing technical advancements targeting sustainability and the circular economy, recovering valuable nutrients from wastewater streams is attracting massive scientific interest. In this context, separating indispensable macronutrients, e.g., nitrogen, from wastewater streams is vital to assure sustainable practices. Typically, techniques developed for such intent are evaluated upon their potential to recover nutrients; however, contaminants of emerging concern existing in these waste-derived nutrient products should not be overlooked [281]. Apart from that, more efficient recycling and reuse routes should be employed in agriculture, especially with the challenges witnessed due to the rising demand for ammonium nitrogen fertilizers and environmental issues associated with their production. Presently, ammonium nitrogen fertilizers in municipal wastewater are anticipated to count for almost 30%. Similarly, the energy and cost of wastewater nitrogen separation and fertilizer production could be potentially reduced through nitrogen recovery from source-separated urine.

The advances witnessed in MCDI have demonstrated it as a potential recovery technique that would back up the sustainability of the nitrogen cycle. For enhancing the electrochemical performance, wide spectrum strategies have been performed via manipulating the operating conditions, and functional groups and by using hybrid systems. For the substantial separation of nitrogen and phosphorus from wastewater, Gao and his research group presented a relatively novel hybrid electrochemical approach comprising MCDI and bipolar membrane ED [282]. According to the authors, almost 77% ammonia and 89% phosphorus were eliminated while 81% of the wastewater effluent was recovered with a water quality high enough to be discharged or reused. Compared to nutrient recovery electrochemical processes, the system enclosed an impressive diminished chemical usage and competitive energy. In another work, the MCDI system was assembled by [283] utilizing a selective ion exchange membrane and mesoporous carbon electrode for ammonium nitrogen removal from wastewater. The work contemplated optimizing three operational conditions, including; the plate spacing, the voltage, and the flow rate. At optimal conditions, the system was able to eliminate about 80% ammonium nitrogen along with other inorganic nitrogen forms such as (90.96%) NaNO_2_, (82.33%) NH_4_Cl and (97.73%) NaNO_3_. Another work reported in the literature applied an anion exchange membrane in the MCDI unit for enhanced nitrate removal. The work tested several membrane functionalizing agents to enhance nitrate separation and electrochemical performance [284]. In a recent study, CDI and MCDI with the aid of copper electrodes were applied for groundwater Denitrification enhancement. The work set forth a potential economical alternative for nitrate removal to gain a more environmentally friendly outcome [285].

The possible synergies of EMCs and other technologies for nitrogen recovery have been demonstrated by several attempts. Tarpeh and coworkers harnessed an electrochemical stripping process that merges ED and membrane stripping for nitrogen recovery from urine. With real urine, the process was capable of recovering 93% of nitrogen while consuming 30.6 MJ kg N^−1^. This is lower than the energy required for conventional ammonia stripping [286]. In another work for ammonia recovery from source-separated diluted urine, a bipolar electrodialysis pilot plant was employed. The plant comprised 3.15 m^2^ of cation exchange membrane and bipolar membrane which revealed that effluent pH set at 4 could bestow more nitrogen removal efficiency (80%) compared to the role of nitrogen load and current density [287].

Examples of nitrogen recovery efficiency and energy requirements with ECMs are given in Table 7. Energy requirement for nitrogen recovery has been reported either per the recovered mass of NH_4_-N (kWh/kg of N) or the volume of the treated waste (kWh/m^3^ of treated waste). The energy figure can be converted from the former to the latter and vice versa if information about flow, nitrogen concentration and its recovery are available. The kWh/m^3^ can be converted to kWh/kg N by dividing it by the product of nitrogen concentration × recovery (%) with special attention to unit conversion. So far, the electrochemical-based membranes have demonstrated promising results when conducted at the lab-scale level and with synthetic feed. However, scaling up these electrochemical systems is crucial and necessitates further investigation. At a large scale, more work is required targeting the operational conditions, investigating new hybrid electrochemical systems, and applying novel routes for process and ion exchange membrane modification. Some successful EMC pilot trials have been reported for streams with low solids such as urine [288], but pilot testing with more complex streams such as domestic and industrial wastewaters should be examined to prove the technique robustness and stability for nitrogen recovery.

## 9. Gas Permeable Membranes (GPMs)

**Figure 13:**
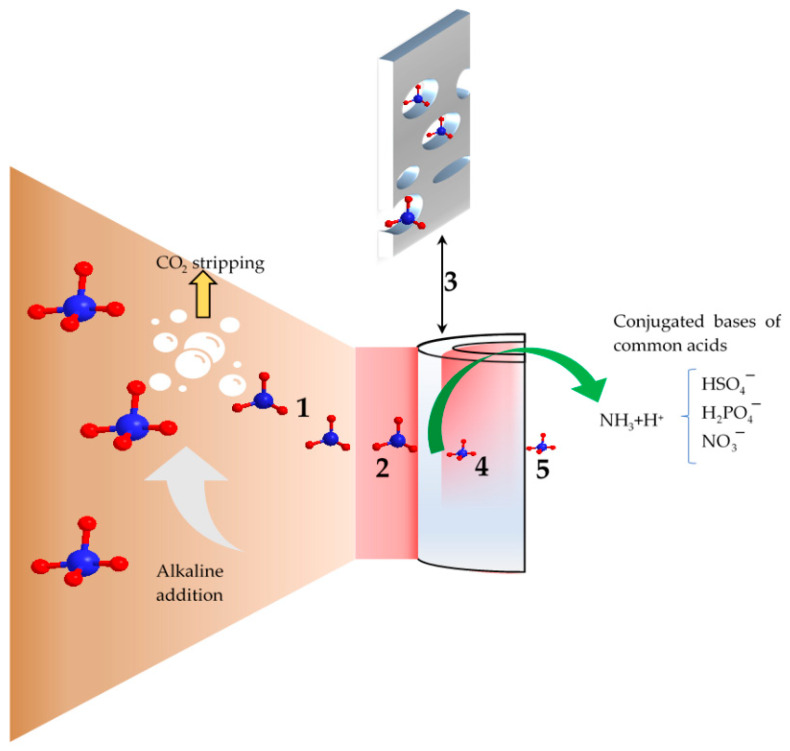
Ammonia recovery principle in membrane contactor.

GMP, also commonly known as membrane contactor, is a technology that utilizes hydrophobic membranes for separating targeted gases, such as ammonia, from liquids. In chemical engineering processes terms, the process is also referred to as transmembrane chemisorption [290]. The working principles of membrane contactors are depicted in Figure 13. The pH of the waste stream is raised to convert NH_4_^+^ to NH_3_. The upper range of pH for the waste stream is around 9 (Table 2), and above this range, a discernible conversion of ammonium to ammonia starts (Figure 8). At room temperature, a 100% conversion is reached at pH = 12. This means that essentially processes/chemicals are required to only raise pH by 3–4 units. However, a pH of 10 was found to be the optimum level for nitrogen recovery from wastewater using membrane contactors [25,26]. Sodium and calcium hydroxides are mostly used for lifting the pH level of wastewater. CO_2_ stripping from wastewater has also been applied for reducing the alkaline needed for raising the pH above 9.4 (pk_a_ of NH_3_) for ammonia recovery [291].

Ammonia transfer from wastewater to the absorption solution consists of five steps as illustrated in Figure 13. First the converted ammonia transfers from the bulk to the boundary layer. This process is driven by the concentration difference. In the second step, the ammonia balances with the air in the pores at the membrane surface. After that, the ammonia diffuses through the membrane pores through either Knudsen diffusion, molecular diffusion, or molecular-Knudsen diffusion depending on the value of the Knudsen number (Kn). It is computed as Kn = λ/d_p_, where λ is the mean free path length for ammonia and d_p_ is the pore diameter [292,293]. If Kn is <0.01, molecular diffusion is the prevailing mechanism. For Kn between 0.01 and 10, the dominant diffusion mechanism is molecular-Knudsen. Knudsen diffusion becomes the governing mechanism when Kn > 10. Considering the quoted value for λ of 76 nm [294] and the typical pore diameter for hollow fibers used in membrane contactors of <0.3 μm, ammonia diffusion is likely to follow the molecular-Knudsen mechanism in most cases. In the fourth step, ammonia reacts with the hydrogen ion donated by the dissociated acid at the membrane surface on the lumen side. Finally, formed ammonium salts transfer from the membrane surface to the acid bulk through concentration difference. Three inorganic acids are commonly used for the chemisorption of ammonia, namely sulfuric acid (H_2_SO_4_), phosphoric acid (H_3_PO_4_), and nitric acid (HNO_3_). These acids are used to produce ammonium salts that can be used as fertilizers. The dissociation of the acids gives different conjugated bases that can bind with ammonium ions forming salts. Ammonium phosphate and ammonium nitrate have higher market values than ammonium sulfate. However, due to the poor dissociation of H_3_PO_4_ at desired pH range for recovery (<7) and the risk associated with handling and storing ammonium nitrate being an explosive chemical, H_2_SO_4_ is commonly selected for ammonia absorption [26].

Membrane contactors can be applied in different configurations; flat sheet, spiral wound, or hollow fibers [72], but the latter is the most popular and feasible configuration. Membrane fibers are normally encapsulated in shells to protect them from mechanical damage. Shells should be carefully packed with membrane fibers to strike a good balance between providing high surface area and maintaining enough space for liquid flow at a reasonable velocity. Overly packed membrane modules may promote fouling and limit their applications to low solids streams. The ideal membrane materials should be hydrophobic, mechanically robust, and thermally and chemically stable [290]. The commonly used membrane materials in the contactor technology are PE, PP, PVDF, and PTFE [294,295,296,297,298]. Among the membrane materials, PTFE exhibited high stability, low fouling tendency, and minimum loss of hydrophobicity [23,299]. The feed solution can run on the shell side or the lumen side of the membrane fibers, and the stripping solution is run on the opposite side of the membrane. The common and more effective way of operating membrane contactors is to run the feed on the shell side since it is the stream with the higher fouling potential [290]. The limited space available on the lumen side may quickly promote membrane fouling. The accessibility into the lumen side is restricted especially when using fine membrane fibers, and this makes physical fouling removal hard. Membrane contactors can operate in open or closed-loop systems depending on the required ammonia removal/recovery level. For high ammonia recovery, close loop operation is applied. However, for the typical wastewater treatment environment, contactors in series design is required to cope with large volumetric flows.

The outcomes of the recent attempts for nitrogen recovery from various waste streams using membrane contactors are summarized in Table 8. In general, membrane contactor technology has achieved high recovery efficiency, especially on a lab scale. The attractive thing about this technology is that the recovered ammonia is converted into high-purity ammonium salts that can readily be used in different industries. It seems that coupling aeration and nitrification inhibitors are a common technique for raising pH in agricultural waste. The aeration applied in the reviewed studies was low to moderate compared to aeration applied in activated sludge, and the pH increase was also slight. Applying nitrification inhibitors should be avoided if the treated stream is directed back to wastewater treatment plants. Also, aeration might be costlier compared to alkaline chemicals addition. Additionally, aeration may exacerbate ammonia loss to the atmosphere if the system was not perfectly sealed. Ammonium salt solutions are produced in large volumes by this technology and need to be concentrated as the transportation of these liquids can incur high costs and have a large carbon footprint. To solve this challenge, a concentrating step can be applied as suggested in Figure 9. Induced crystallization can also be utilized to harvest ammonium salt crystals during the operation as proposed in [292]. The study of Davey and co-workers [292] warned that the antisolvents used for instigating crystallization should carefully be selected. These antisolvents should not affect membrane surface characterization, mainly not to lower the surface tension of acid solution which can lead to membrane wetting and leaching of feed solution.

Fouling and wetting are the two major challenges in membrane contactor technology. Fouling can be minimized by applying high feed flow to induce turbulences adjacent to the membrane surface which in turn reduces foulant adherence. In any case, chemical membrane cleaning is inevitable after long operational periods. Diluted acid and alkaline solutions as well as enzymes have been used for membrane cleaning [300]. Although NaOH was found to be effective in removing surfactants (potential wetting causing foulants) [301], it can expedite membrane aging [300]. Enzymes appear to be less effective than NaOH [302]. However, from the authors’ personal experience of pilot testing with different streams namely urine, digester centrate, and landfill leachate, fouling had an insignificant effect on ePTFE membrane hydrophobicity and performance, and washing with diluted H_2_SO_4_ was enough to restore membrane properties. Manufacturing superhydrophobic membranes can be another approach to tackle fouling and wetting [303,304]. Coating with a hydrophilic layer to attract the hydrophilic end of surfactants and result in a hydrophobic outer surface or incorporating nanoparticles for increasing membrane roughness are other interesting ways that can be applied in membrane contactor technology [304,305].

## 10. Effective Hybrid Membrane Systems for Nitrogen Recovery

**Figure 14:**
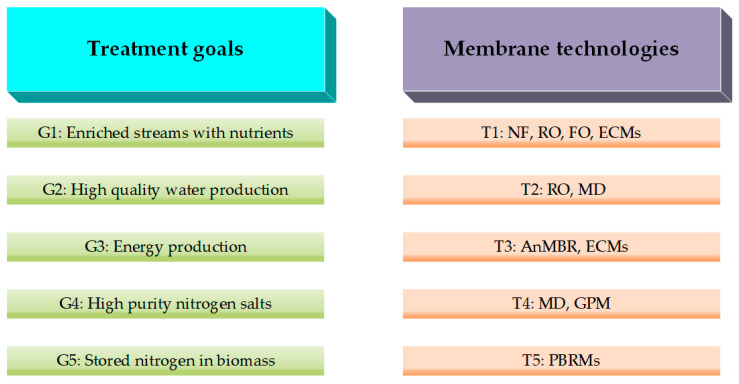
Membrane technology selection matrix based on the recovery goals.

The discussions in the previous sections highlighted the fact that all membrane technologies have some shortcomings that hinder their capacity as a standalone nutrient recovery solution. Hence, harnessing the synergy between the various membrane-based technologies was found to be an effective strategy for producing processes with high nutrient recovery efficiency [3]. This section aims to present a roadmap for the selection of suitable hybrid membrane systems for nitrogen recovery by clearly defining the goals of the process. Nitrogen recovery is linked to the recovery of other resources such as energy, pure water, and phosphorous. However, phosphorous recovery is beyond the scope of this work and it has not been considered in the evaluation of the recovery technologies. The other aspect that should be considered is the quality of the recovered products. Hence, based on the literature and the authors’ knowledge of the subject matter, we have identified five treatment goals associated with nitrogen recovery along with specifying the membrane technologies that can fulfill such goals as illustrated in Figure 14.

Some technologies can achieve two goals, such as RO, MD, and ECMs. The product of G1 has limited applications as an enriched nutrient waste stream that might be used for direct spreading onto the agricultural fields but they must meet stringent regulatory limits. The better utilization for the enriched streams then is the use as a feed for subsequent recovery processes. For G5, only T5 satisfies this goal, and no other technologies. In addition to energy saving associated with nitrogen removal, technologies in T2, T3 and T4 groups can generate marketable products. For maximizing the benefits of membrane hybrid systems, special attention should be given to these technologies. That is not to ignore the benefits that can be reaped from combining technologies from T1 and T5. Such combinations were proven to be feasible for specific scenarios such as combining FO with PBRMs for treating a small-scale facility [313].

The performance and energy figures for individual and hybrid systems of technologies in T2–T4 groups are provided in Table 9. It should be noted that studies for other hybrid systems also exist, but energy discussions were omitted in these studies and hence they have been excluded. Based on energy requirements and nitrogen recovery, GPM seems to be the most efficient option as a standalone technology. Additionally, GPM and RO are the most tested technologies on a pilot scale with long-term runs, and this reflects their maturity. However, GPM pilot trials have been shown to require higher energy and produce less nitrogen recovery compared to lab studies. This is expected as pilot runs in a real industrial environment are often not optimized. Recent studies have also highlighted the potency of GPM as an attractive nitrogen recovery technique. Beckinghausen et al. [1] conducted a thorough analysis of a wide range of nitrogen recovery techniques (membrane-based and non-membrane based) and concluded that GPM is the most promising nitrogen recovery technology for future investment. For the same goal, but using a different approach, Munasinghe-Arachchige and Nirmalakhandan [314] applied a multi-criterion decision-making tool using the PROMETHEE method for evaluating and ranking common nitrogen recovery techniques. The techniques compared were air stripping as the industry standard, UF/ion exchange and UF/RO as mature pressure-driven membrane technologies, struvite precipitation as a mature chemical recovery technique, and GPM as a promising low-energy membrane technique. They used ten criteria in their evaluation: efficiency, energy for pre-treatment, energy for removal, chemical requirement, the cost for chemicals, N content in the recovered product, price of fertilizers, profit, number of steps, and purity of the products. Such evaluations are valuable as they capture the process cost directly and the environmental impact indirectly by identifying energy and chemical demands. GPM was ranked the highest due to low energy demands and the high content and purity of nitrogen products. GPM has been combined with other technologies mostly with ECMs to harness the ammonium accumulation and the pH rise at the cathode compartment. Although reported energy requirements for some GPM + ECMs hybrid system was low, they were only tested on a lab scale and the nitrogen recovery achieved was low. A promising combination that has not been reported in the literature is GPM and AnMBR. The stream coming from AnMBR has less carbon which means low fouling potential and less competition with NH_3_ given that methane is removed before entering the GPM system. RO and induced crystallization can be used to produce pure ammonium salts from the stripping solution.

RO technology alone or combined with other technologies, such as AnMBR, requires moderate energy, but the product is of low quality. The energy requirement for AnMBR presented in Table 7 was obtained based on the modeling of the process rather than actual testing. An MBR designed for a typical wastewater treatment process requires low energy of 0.1–0.15 kWh/m^3^ [315]. The higher end of the energy range of 5.7 kWh/m^3^ is due to the inclusion of aeration needed for reducing fouling formation on membranes. In some cases, only the generated energy of the AnMBR is reported as is the case for Prieto et al. [316] who obtained 95.5% cumulative nitrogen recovery with a biogas production of 4.5 L/d. MD in its different configurations requires high energy, and its application is only feasible when access to low-grade energy or other resources is available. Although the concept of an isothermal membrane was proposed in the literature as an energy-saving approach with nitrogen recovery of ~60% using ~2.2 kWh/kg N [317]. We are not sure if we can consider this approach as MD as the operation principles resemble very much that of the GPM. A recent numerical study showed that SGMD can be used for high nitrogen recovery (99%) with relatively low energy requirements of 1.42 kWh/m^3^ feed. This study suggested utilizing air with high relative humidity for reducing water vapor transfer and maximizing ammonia recovery. This idea can be harnessed in coastal areas where the air already has high relative humidity. MFC and MEC have been selected as examples of ECM technologies due to energy generation with these technologies and their common use. These two technologies require moderate energy levels, but the products are concentrated nitrogen streams that require further processing. It should be noted that MEC has a higher nitrogen recovery compared to MFC due to the additional power applied that induces ammonia transfer from the anode compartment to the cathode compartment [11]. For MFC, there is a conflict between electrical energy recovery and nitrogen recovery. For recovering high energy, moderate electrical power should be applied and this negatively impacts ammonia transfer and accumulation in the catholyte [3]. The recovery in some MEC + GPM systems might seem to be small as it is the case in [288], but this is because part of the nitrogen was already consumed in the phosphorus recovery step for producing struvite in the proceeding processes. The other reason for this low ammonia recovery is that the pH level in the catholyte is ≤pKa of ammonia (9–9.5) where ~50% of recoverable nitrogen is in the form of NH_4_^+^. This means that there is a need for raising the pH through alkali addition, but this increases the cost of the process further. The reactions induced by the electrical current in the ECMs process can be a challenge, especially with streams that contain high amounts of inorganic elements, such as urine. It was found that the produced chlorine from electrochemical oxidation of urine can react with ammonium forming chloramine, or forms hypochlorite that oxidizes ammonium to nitrogen, both of which can impair nitrogen recovery [318]. The performance of MFC + OMBR is impaired by ammonium ions transfer to balance the charge on the draw solution side caused by the reverse salt flux of cations. Based on the evaluation of the technologies in T2–T4 groups and their reported combinations, GPM seems to be the technology that is likely to be seen applied on an industrial scale. The liquid ammonium salts can be marketed as products for fertigation and hydroponics applications to reduce the cost associated with concentration and drying steps.

## 11. Outlook for Realistic Research Development

The progress of nitrogen recovery research should be guided by the industry’s need for both nitrogen removal and the use of the recovered products with a focus on scalability. The main sources of nitrogen-rich waste streams are domestic wastewater and livestock farms. When it comes to domestic wastewater, the argument of centralized or decentralized treatment systems comes up as the latter produces more concentrated streams and this makes the recovery process economically more efficient [305]. However, the viability of implementing decentralized wastewater treatment systems from infrastructure investment and maintenance perspectives needs to be answered before considering it for nitrogen recovery on a large scale. The status quo of domestic wastewater is centralized and that is where the focus should be. Domestic wastewater is the biggest nitrogen source and recovery processes should cater to this stream. The main goal of wastewater treatment plants is to remove pollutants and nutrients to low levels that meet the regulatory limits. So, the evaluation of recovery technologies in terms of nitrogen removal and the retention time of the process should be benchmarked against those of wastewater treatment plants. The recovery technologies should also be capable of coping with wastewater quality fluctuations, and this can be examined through long-term testing. Most membrane-based nitrogen recovery studies are still based on small-scale trials in a controlled environment, while the focus should be on the transition of new and existing techniques from small to pilot and then large scales. This gives confidence to the industry to invest in these technologies and consider them in their future development plans. Digester reject water seems to be the best line for nitrogen recovery in wastewater treatment plants due to its high nitrogen content. Since this line is recycled back to the treatment train, studies that alter the physicochemical properties of wastewater, such as ECMs, should explore the effect of this alteration on the microbial communities of the activated sludge. For farm wastewater, developing membrane systems capable of selectively removing heavy metals and micropollutants would be the most practical and cost-effective solution as this enables farmers to safely spread waste onto their lands.

Understanding the requirements of the end users of the recovered product is of great importance for the evolution of this research subject. Recovered nitrogen has very much been linked to agricultural applications. While agriculture is the biggest sector that can benefit from recovered nitrogen as alternative fertilizers, exploring the need of other industries for such materials is important. This may increase recovered product values and allow more flexibility when it comes to product quality. The farming needs for recovered nitrogen products are not appropriately understood as was rightly pointed out in [1]. Most of the nitrogen fertilizers used are urea-based followed by ammonium nitrate, whereas most of the recovered nitrogen was in the form of ammonium sulfate. Urea instability and poor rejection with membrane technologies are what ruled it out from recovery considerations with membrane technologies. However, investigating efficient ways for producing crystalline ammonium nitrate and phosphate from recovered nitrogen with high purity is of utmost importance. Understanding the interaction between ammonia and different membrane materials can help in producing membranes with high selectivity for ammonium rejection or ammonia gas transfer. Modeling and artificial intelligence tools can also be used to speculate the performance of different newly developed technologies on a large scale using literature and industry data for training the models.

## 12. Conclusions

This study provided a critical analysis of the literature body pertaining to nitrogen recovery from waste using scientometric analysis. Four main research themes have been identified namely: membrane technologies for recovery purposes, biological processes, energy recovery, and nitrogen sources. Membrane technologies appeared to occupy a decent share of nitrogen recovery research work. Research into nitrogen recovery evolved from the early nineties where the focus was nutrient concentration for spreading on agricultural fields to more sophisticated approaches for extracting nitrogen in high-purity products using advanced membrane designs. A thorough review and discussions of the different membrane technologies studies are also presented with a focus on recent literature. Existing conceptual designs of some membrane processes were scrutinized and new designs were proposed for more efficient and resilient processes. In general, nitrogen can be recovered from waste streams either as enriched streams, high-purity ammonium salts, or in the form of biomass (e.g., algae). Pressure- and osmotic-driven membranes as well as electro- and biologically-enhanced membranes produce enriched nitrogen streams. Thermally-driven membranes and GPM produce ammonium solutions with high purity. Nitrogen and carbon-rich biomass can be obtained from PBRM processes. Membrane technologies as standalone or hybrid systems that produce marketable nitrogen products along with energy and high-quality water are likely to be considered for large-scale implementation. Among all membrane recovery technologies, GPM is the most promising one. Hybrid systems based on GPM suggested in the literature and proposed in this study are likely to break through the scalability barrier and reach industrial implementation before other technologies. More attention should be paid to the practicality of the recovery technologies and the needs and priorities of the wastewater industry and the potential prospective customers of the recovered nitrogen products should always be kept in mind.

## Figures and Tables

**Table 1:** Thesaurus components for keywords occurrence analysis.

Originally Captured Keywords	Replacement
Ammonia capture	Nitrogen recovery
Ammonia recovery	
Ammonia removal	
Ammonium recovery	
Ammonium removal	
nitrogen recovery	
nitrogen removal	
nutrient recovery	
nutrient removal	
nutrients recovery	
nutrients removal	
microbial fuel cell	microbial fuel cell
microbial fuel-cell	
microbial fuel-cells	
reverse osmosis	Reverse Osmosis
reverse-osmosis	
bioreactor	Bioreactor
bioreactors	
fuel-cell	Fuel-cell
fuel-cells	
bioreactor	Bioreactor
bioreactors	
gas-permeable membrane	Gas-permeable membrane
gas-permeable membranes	
MBR	Membrane bioreactor
membrane bioreactor	
membrane contactor	Membrane contactor
membrane contactors	
membrane technology	Membrane technology
membranes	
municipal waste-water	Wastewater
municipal wastewater	
waste-water	
wastewater	
wastewaters	
sewage treatment	Sewage treatment
sewage-treatment	

**Table 2:** Literature of examples of waste streams for nitrogen recovery.

Waste Stream	N Concentration (g/L)	Solids Content (g/L)	Other Characteristics			Ref.
			pH	COD (g/L)	Conductivity (mS/cm)	
Domestic Wastewater	0.025–1.2	0.2–0.8	6.5–8,	0.17–0.9	1.2–18	[17,27,29,37,40,77,78,79,80,81,82]
Urine	0.2–8.5	0.25–0.32	6–11	1–20	26–50	[42,43,83,84,85,86]
Manure slurry	1–5.5	0.5–15	7–9	5–45	1–24	[19,87,88,89,90,91,92,93,94,95,96,97,98]
Source separated black water	0.12–1.2	0.2–1.6	7–9	0.5–8	1.9–8	[60,99,100,101,102,103]
Food waste *	4–3	13–45	4–5.5	73–160 **	7.5–9.5	[63,64,104,105,106,107]
Aquaculture wastewater	0.0003–0.016	0.001–0.08	6–8	0.008–0.14	0.8–2.3	[68,69,108,109]
Slaughterhouse wastewater	0.030–0.2	0.2–0.45	6–7.5	0.25–11.5	1–4	[110,111,112,113,114]
Landfill leachate	1–4.5	0.025–9	7.8–8.5	1.5–10	2.5–28	[10,74,76,115,116,117]

* Characterizations presented in g/kg, ** The value represents the total COD.

**Table 3:** Summary of nitrogen recovery with pressure-driven membranes.

Membrane Technology	Membrane Material	Feed Type	Flux (LMH)	NH_4_^+^ Rejection (%)	Ref.
MF	Aluminum oxide (Al_2_O_3_) ceramic membranes	Filtered sow slurry (15–20 g/L)	-	Slurry volume reduced by 70%	[19]
	α-Al_2_O_3_ flat sheet ceramic membranes	Pre-coagulated domestic wastewater	41.7	40–50	[132]
	PVDF	Pre-coagulated raw sewage	-	28–52 *	[133]
	PVDF	Raw sewage	5–10	37.5	[79]
UF	PVDF	Pig manure after settling tank	~9 *	Concentration factor of 3.7	[89]
		Pig manure after screening	~7 *	Concentration factor of 3.7	
		Pig manure after screening + settling	~10 *	Concentration factor of 2.2	
		Pig manure after screening + settling + aerobic bioreactor	~34 *	Concentration factor of 4.3	
	Multi-channel membrane with an active surface layers made of Al_2_O_3_, TiO_2_ and ZrO_2_	Laundry wastewater	~130 *	~99	[134]
	PVDF	Primary clarifier effluent	91–168 depending on flow velocity and applied pressure	Only 10% rejection of total N but nor rejection of NH_4_^+^	[135]
	PVDF	screen raw sewage with 0.56 mm sieve	70–110 depending on flow velocity and applied pressure	Only 10% rejection of total N but nor rejection of NH_4_^+^	
	PVDF	Activated sludge effluent	~14 *	0–58% rejection when varying filtration/backwash time ration of 5–9	[136]
NF	PA active layer+ PSU support layer	Dairy manure digestate	125–150 at pH = 11	~32	[137]
	PA active layer+ PSU support layer	Synthetic urine	~130–170 for pH = 3–9	45 *	[138]
	PES	Aquaculture effluent	~8–18 for 2–10 bars	68	[139]
	PA	Synthetic wastewater with micropollutants	-	60	[140]
RO	PA	Anaerobically digested pig manure	~10–68 * depending on the concentration factor achieved	95	[141]
	PA active layer+ PSU support layer	Prefiltered Sow slurry with MF	-	~91 *	[19]
	PA	Prefiltered heifer wastewater by 30 μm filter	30	96	[92]
	PA	Pre-treated swine manure with diatomaceous earth	-	~98 for pH 4.5–7	[96]
	PA	Pre-filtered swine manure	-	66.6	[94]
	PA	Pre-filtered swine manure	~30 *	Concentration factor of 5.6	[93]
	RO (Dow, USA)	Pre-treated digested cattle manure with screw press separation + centrifugation + UF	-	99.5	[142]
		Pre-treated digested swine manure with screw press separation + centrifugation + UF	-	96	
	PA	Effluent of fluidized bed reactor + anaerobic membrane bioreactor	12.3	94–100 for pH = 8–4	[29]
	PA	Municipal wastewater	~52 *	~100	[143]

* Estimated from figures or provided information.

**Table 4:** Nitrogen recovery with MD technologies.

Configuration	Membrane Material	Feed Type	NH_4_^+^ Rejection (%)	Ref.
DCMD	PTFE	palladium leachate	97.4	[158]
Modified DCMD by solar energy system	PP	landfill leachate	59	[159]
DCMD with acid absorption	PVDF	Synthetic NH_4_Cl solution	99.5	[146]
DCMD with acid absorption	PTFE	Ion exchange brine	>96	[160]
DCMD	Nafion ionomer and Multiwall Carbon Nanotubes (MWCNTs)+ a Poly (vinylidene fluoride-cohexafluoropropene; PVDF-HFP)	Sludge digestate	~5–60 for pH = 7–12	[24]
DCMD	PTFE with PP scrim backing	Synthetic ammonia solution	90	[161]
VMD	PP	Biogas slurry	98	[162]
VMD	PP	Synthetic solution of NH_4_OH	Concentration factor of ~10–15	[163]
VMD	PTFE	Synthetic solution of NH_4_OH	90	[164]
VMD	PTFE	Liquid digestate	~95	[165]
VMD	PTFE	Simulated wastewater made of NH_4_Cl, Na_2_CO_3_ and Na_2_SO_4_	93.3 at pH = 4	[166]
Two stages DCMD	PP	Anaerobic digestion effluent	~81	[167]
VMD	PTFE	Human urine	40–75	[168]
VMD	PTFE	Biogas slurry	Concentration factor of 8	[169]
SGMD	PTFE	Synthetic ammonia solution	97	[148]
Membrane bioreactor (MBR) + DCMD	PVDF	Synthetic NH_4_Cl solution	76–94	[170]
MBR + DCMD	PTFE	Anaerobic effluent	89.6–96.3	[171]

**Table 5:** Literature summary for nitrogen recovery with FO process.

Membrane Materials & Orientation	Waste Stream	Draw Solution	Operational Mode	NH_4_^+^ Rejection (%)	Flux	Ref.
Flat sheet CTA, asymmetric	Spiked activated sludge with glucose, NH_4_Cl and K_2_HPO4	NaCl	FO	96	2.5–6.15	[211]
Flat sheet CTA, symmetric	Synthetic hydrolyzed urine	NaCl	FO	50–80	10–24	[212]
Flat sheet CTA with nonwoven support layer, asymmetric	Synthetic secondary treated wastewater effluent	MgCl_2_	FO	99.4	~10	[213]
Spiral-wound CTA	Real domestic wastewater	NaCl	FO	48	6	[214]
Flat sheet CTA embedded in polyester mesh support, asymmetric	MBR effluent	Synthetic sea water	FO	Concentrated by 2.1-fold	~4.8–5.5	[189]
Flat sheet CTA embedded in polyester mesh support	Anaerobic acidogenic fermentation of anaerobic MBR	NaCl	FO	92–97 for pH = 3–7	~14	[215]
Flat sheet CTA embedded in polyester mesh support	Real municipal wastewater	NaCl	FO	93	Initial flux varied: ~8–25 with draw solution concentration change of 0.5–4 M	[210]
Flat sheet CTA embedded in polyester mesh support	NH_4_Cl dissolved in background electrolyte solution (10 mmol/L NaCl + 0.1 mmol/L NaHCO_3_)	NaCl	FO	4.5–78 for water flux of ~3.5–18 LMH	~3.5–18 for NaCl concentration of 0.25–3.0 mol/L	[216]
Flat sheet CTA	Centrate of digested swine wastewater	MgCl_2_	FO	NH_4_^+^ penetration was desirable rather than rejection (93% of NH_4_^+^ passed through)	Maximum of 3.1	[217]
Flat sheet CTA	Anaerobically treated palm oil mill effluent	(NH_4_)_2_SO_4_	FO	Concertation factor of 0.7	~2.1	[218]
		NH_4_H_2_PO_4_		Concertation factor of 1.65	~2.6	
		KCl		Concertation factor of 1.8	~1.9	
Flat sheet polyamide TFC	NH_4_Cl dissolved in background electrolyte solution (10 mmol/L NaCl + 0.1 mmol/L NaHCO_3_)	NaCl	FO	<10 for all tested water fluxes	~11–32 for NaCl concentration of 0.25–3.0 mol/L	[216]
Flat sheet TFC	Treated sewage effluent	NaCl + (NH_4_)_2_HPO_4_	FO	97	~13	[219]
Flat sheet TFC	Treated sewage effluent	NaCl + (NH_4_)_2_HPO_4_	PRO	95	~10.5	[219]
Flat sheet TFC, symmetric	Synthetic municipal wastewater	Synthetic seawater	FO	67	12	[220]
Virgin polyamide (PA) TFC	Synthetic ammonium solutions	MgCl_2_	FO	~97	0.7	[221]
Virgin polyamide (PA) TFC	Secondary return activated sludge	MgCl_2_	FO	75.5	~0.6	[221]
Surface modified PA TFC (grafted with 3% polyethylenimine (PEI))	Synthetic ammonium solutions	MgCl_2_	FO	100	~1.3	[221]
Surface modified PA TFC (grafted with 1.5% polyethylenimine (PEI))	Secondary return activated sludge	MgCl_2_	FO	~89	~0.3	[221]
Aquaporin Inside™ TFC flat sheet	Centrate of cow manure digestion	NaCl	FO	~95	~7.5	[222]
Aquaporin Inside™ TFC flat sheet	Centrate of cow manure digestion	Hide preservation wastewater	FO	~95	~6.3	[222]
Aquaporin Inside™ TFC flat sheet	Anaerobic digester effluent	MgCl_2_	FO	~97	~2–3.3	[223]
Aquaporin Inside™ TFC flat sheet	Sewage	MgCl_2_	FO	66	5.3	[224]
Aquaporin A/S TFC flat sheet	Centrate of digested swine farm	NaCl	FO	~40	Water flux varied between ~6 and ~4 for water recovery of 10% and 50%, respectively	[202]
Flat sheet TFC	Centrate of digested swine farm	NaCl	FO	~45	Water flux varied between 6 and ~1.5 for water recovery of 10% and 50%, respectively	[202]
PA flat sheet TFC	NH_4_Cl	NaCl	FO	~40	~14	[225]
PA flat sheet TFC grafted with quaternized polyethyleneimine	NH_4_Cl	NaCl	FO	~95	~6.5	[225]

**Table 6:** Summary of recent literature for nitrogen recovery with FO process.

OMBR Configuration	Waste Stream	Draw Solution	NH_4_^+^-N Rejection (%)	Ref.
Submerged aerobic OMBR (plate and frame)	Synthetic domestic wastewater	NaCl	90	[248]
Submerged aerobic OMBR (plate and frame)	Synthetic domestic wastewater	NaCl	>60	[240]
Submerged aerobic OMBR (FO cell)	Synthetic wastewater	NaCl	70–80	[249]
Submerged aerobic OMBR (plate and frame)	Activated sludge	NaCl	97	[243,250]
Submerged aerobic OMBR (plate and frame) with UF membrane	Activated sludge	NaCl	>80	[251]
Side-stream OMBR (plate and frame)	Activated sludge	NaCl	98	[252]
Submerged anoxic OMBR (plate and frame)	Synthetic wastewater	NaCl	68	[253]
Anaerobic submerged OMBR tubular module with MF membrane and moving sponge	Real domestic wastewater	A mixture of Na_3_PO_4_ and EDTA	75	[246]
Anaerobic submerged OMBR tubular module with UASB	Anaerobic granular sludge	MgSO_4_	55–86	[247]

**Table 7:** Nitrogen recovery and energy requirements of ECMs.

Process	Wastewater Stream	Nitrogen Recovery	Energy Consumption	Ref.
bipolar electrodialysis (pilot plan)	source-separated diluted urine	88% of the NH_4_^+^	13 Wh/gN	[287]
microbial fuel cell (bio-electrochemical system)	mariculture wastewater	94.05% NH_4_^+^-N and 77.35% inorganic nitrogen	-	[279]
combination of electrodialysis and membrane stripping	source-separated urine	93% ammonium sulfate	30.6 MJ kg N^−1^	[286]
carbon electrode–based membrane capacitance deionization	Simulated wastewater	82.33% NH_4_^+^, 90.96% NO_2_^−^, and 97.73% NO_3_^−^	-	[283]
membrane capacitive deionization (pilot-scale)	Municipal wastewater	39.12 ± 5.31% Ammonia	1.16 kWh/m^3^	[289]
enhanced Flowed Channel Membrane Capacitive Deionization	digestate wastewater	89% and 67% with synthetic and real digestate wastewater^+^	-	[282]

**Table 8:** Summary of recent studies of nitrogen recovery with membrane contactor.

Membrane Type	Waste Stream	pH Raising Agent	Stripping Solution	NH_4_^+^-N Recovery (%)	Scale	Ref.
Expanded polytetrafluoroethylene (ePTFE)	Diluted swine manure	Aeration+NaHCO_3_+N-Allylthiourea (nitrification inhibitor)	H_2_SO_4_	99	Lab	[31]
ePTFE	The effluent of anaerobically digested swine wastewater	Aeration+nitrapyrin (nitrification inhibitor)	H_2_SO_4_	96–98	Lab	[306]
ePTFE	Swine manure centrate	Aeration+Allylthiourea	H_2_SO_4_	90	Lab	[307]
ePTFE	Raw manure and digestate of dairy farm	-	H_2_SO_4_	~3–13	Lab	[308]
PP	Centrate of anaerobic digester of domestic wastewater treatment	NaOH	H_2_SO_4_	>99	Lab	[25]
ePTFE	Treated mesophilic digester wastewater with ballasted sedimentation	Slaked lime	H_2_SO_4_	55	Pilot	[26]
			HNO_3_	41		
			H_3_PO_4_	48		
ePTFE	Treated mesophilic digester wastewater with ballasted	Slaked lime	H_2_SO_4_	92.5	Pilot	[23]
	Landfill leachate	NaOH		86.5		
	Human urine	Slaked lime		70		
PP	Synthetic hydrolyzed human urine	KOH	H_3_PO_4_	90.9	Lab	[309]
PP	Hydrolyzed human urine	NaOH	H_2_SO_4_	93	Lab	[310]
PP	Synthetic hydrolyzed human urine	NaOH	H_3_PO_4_	97	Lab	[311]
PTFE	Hydrolyzed urine	Ca(OH)_2_	H_2_SO_4_	98	Lab	[312]

**Table 9:** Summary of energy requirements and performance of single and hybrid systems from technologies in T2–T4 groups as reported in the literature.

System		Waste Stream	Energy Requirements	NH_4_^+^-N Recovery/Concentration	Scale	Ref.
RO		Animal manure	4.3–5.5 kWh/m^3^ feed	90–98% retention	Pilot	[127]
MD	VMD	Biogas slurry	9.5–32.6 kWh/m^3^ feed *	87%	Lab	[169]
	DCMD	Anaerobic digester effluent	8.6–45.4 kWh/m^3^ feed *	>98%	Lab	[167]
	SGMD	Simulated centrate of domestic wastewater digested	1.42 kWh/m^3^ feed *	99%	Modeling	[319]
	AGMD	Filtered hydrothermal liquefaction wastewater with UF	16.6 kWh/m^3^ feed ^◊^	Obtained 80% water recovery with an NH_4_^+^ concentration of 13 g/L	Lab	[320]
AnMBR		Domestic wastewater	0.03–5.7	-	Modeling	[238]
ECMs	MEC	Synthetic wastewater and simulated anaerobic digestion effluent	1.3–4.0 kWh/kgN	90–94%	Lab	[274,321]
	MFC	Urine	1.86 kWh/kgN	0.32g N/d.m^2^	Lab	[322]
GPM		Digested and centrifuged animal manure, and influent of anaerobic digester	0.17–1.2kWh/kgN ^§^	90–98%	Lab	[306,307,308,323,324]
		Landfill leachate and urine	8.8–11.4 kWh/m^3^ feed	70–86.5	Pilot	[23]
Hydrogen recycling electrochemical system (HRES) + GPM		Urine	8.5 kWh/kgN with a current intensity of 10 A/m^2^	64	Lab	[325]
			7.3 kWh/kgN with a current intensity of 20 A/m^2^	73		
			15.7 kWh/kgN with a current intensity of 50 A/m^2^	60		
MEC + GPM		Urine	5.6–13.8 kWh/kgN	90	Lab	[318]
MEC + GPM		Urine	1.4 kWh/kgN	31	Pilot	[288]
		Urine	2.5 kWh/kgN	49	Lab	[326]
MEC + Ion exchange membrane		Urine	1.8 kWh/kgN	Ammonia concentrated by a factor of 4.5	Lab	[327]
MEC + AnOMBR		Synthetic wastewater	40 kWh/kgN ^§^	45	Lab	[328]
MEC + GPM		Synthetic influent black water	9.7 kWh/kgN	83	Lab	[329]
Bipolar MEC + GPM		Synthetic wastewater	0.76 kWh/kgN	65	Lab	[330]
Capacitive deionization membrane+ GPM		Synthetic wastewater	9.9–21.1 kWh/kgN	60	Lab	[331]
MEC + GPM		Synthetic wastewater	8.12–11.9 kWh/kgN	9–74	Lab	[332]
MFC + OMBR		Synthetic wastewater	1.23 kWh/m^3^ feed ^#^	Concentrated NH_4_^+^ from 20 to 30 mg/L at the start and then went back to 20 mg/L	Lab	[333]
MEC + OMBR		Synthetic wastewater	1.23 kWh/m^3^ feed ^#^	72–78.5	Lab	[334]
RO + AnMBR		Domestic wastewater	3–6 kWh/m^3^ feed	>90% concentrated N	Pilot	[20]
RO + AnMBR		Domestic wastewater	3–7 kWh/m^3^ feed	>90% concentrated N	Pilot	[335]

* Energy was calculated based on the cost/volume of the treated waste stream and the price of electricity, ^◊^ calculated based on the methods presented in [336], ^§^ reported figures are based on calculations done by [1,314], ^#^ based on the energy figures reported in [246].

## Data Availability

Not applicable.
